# Supplementary material for: Why ‘one size fits all’ is not enough when designing COVID-19 immunity certificates for domestic use: a UK-wide cross-sectional online survey
Source: BMJ Open. 2022 Apr 15;12(4):e058317. doi: 10.1136/bmjopen-2021-058317 (PMC9013794; doi:10.1136/bmjopen-2021-058317)
Supplement: Supplementary data [file bmjopen-2021-058317supp001.pdf]

Supplementary Materials

Supplementary Table 1 Demographic characteristics of sample

		Freq.	Percent	Cum.
**Gender**	Female	277	51.87%	51.87%
	Male	254	47.57%	99.44%
	Self-define	1	0.19%	100%
	Prefer not to say	2	0.37%	99.81%
**Age**	18 – 23	77	14.42%	14.42%
	24 – 29	51	9.55%	23.97%
	30 – 39	95	17.79%	41.76%
	40 – 49	87	16.29%	58.05%
	50 – 59	95	17.79%	75.84%
	60 – 69	109	20.41%	96.25%
	70 or older	20	3.75%	100%
**Ethnicity**	Asian	34	6.37%	6.37%
	Black	20	3.75%	10.11%
	Hispanic/Latino	3	0.56%	10.67%
	Mixed	15	2.81%	13.48%
	Other	8	1.50%	14.98%
	South Asian	12	2.25%	17.23%
	White	442	82.77%	100%
**Region**	East Midlands	42	7.87%	7.87%
	East of England	35	6.55%	14.42%
	London	81	15.17%	29.59%
	Northeast	32	5.99%	35.58%
	Northern Ireland	11	2.06%	37.64%
	Northwest England	58	10.86%	48.50%
	Scotland	37	6.93%	55.43%
	South-East England	87	16.29%	71.72%
	Southwest of England	43	8.05%	79.78%
	Wales	19	3.56%	83.33%
	West Midlands	45	8.43%	91.76%
	Yorkshire and the Humber	44	8.24%	100%
**Area**	Rural	166	31.09%	31.09%
	Urban	368	68.91%	100%
**Accommodation**	Living alone	87	16.29%	16.29%
	Living in shared accommodation	54	10.11%	26.40%
	Living with other family members	382	71.54%	97.94%
	Other	11	2.06%	100%
**Employment**	Employed/Self-employed	340	63.67%	63.67%
	Retired	97	18.16%	81.84%
	Unemployed	97	18.16%	100%
**Education**	A level (or equivalent)	130	24.34%	24.34%
	GCSE (or equivalent)	80	14.98%	39.33%
	Postgraduate degree	95	17.79%	57.12%
	Undergraduate degree	175	32.77%	89.89%
	Vocational	54	10.11%	100%
**Disability**	No	467	87.45%	87.45%
	Prefer not to say	6	1.12%	88.58%
	Yes	61	11.42%	100%
All		*534*	100%	
