# Supplementary material for: Why ‘one size fits all’ is not enough when designing COVID-19 immunity certificates for domestic use: a UK-wide cross-sectional online survey
Source: BMJ Open. 2022 Apr 15;12(4):e058317. doi: 10.1136/bmjopen-2021-058317 (PMC9013794; doi:10.1136/bmjopen-2021-058317)
Supplement: Author's manuscript [file bmjopen-2021-058317.draft_revisions.pdf]

BMJ Open is committed to open peer review. As part of this commitment we make the peer review history of every article we publish publicly available.

When an article is published we post the peer reviewers' comments and the authors' responses online. We also post the versions of the paper that were used during peer review. These are the versions that the peer review comments apply to.

The versions of the paper that follow are the versions that were submitted during the peer review process. They are not the versions of record or the final published versions. They should not be cited or distributed as the published version of this manuscript.

BMJ Open is an open access journal and the full, final, typeset and author-corrected version of record of the manuscript is available on our site with no access controls, subscription charges or pay-per-view fees (http://bmjopen.bmj.com).

If you have any questions on BMJ Open's open peer review process please email info.bmjopen@bmj.com

BMJ Open

Why "one size fits all" is not enough when designing COVID-19 immunity certificates for domestic use: a UK wide cross-sectional online survey

Journal:	*BMJ Open*
Manuscript ID	bmjopen-2021-058317
Article Type:	Original research
Date Submitted by the Author:	26-Oct-2021

Complete List of Authors:	Niculaescu, Corina; Brunel University London, Department of Computer Science<br>Sassoon, Isabel; Brunel University London, Department of Computer Science<br>Landa-Avila, Irma; Loughborough University<br>Colak, Ozlem; Loughborough University<br>Jun, Gyuchan; Loughborough University, Loughborough Design School<br>Balatsoukas, Panagiotis; Loughborough University

Keywords:	Public health < INFECTIOUS DISEASES, COVID-19, PUBLIC HEALTH

SCHOLARONE™
Manuscripts

I, the Submitting Author has the right to grant and does grant on behalf of all authors of the Work (as defined in the below author licence), an exclusive licence and/or a non-exclusive licence for contributions from authors who are: i) UK Crown employees; ii) where BMJ has agreed a CC-BY licence shall apply, and/or iii) in accordance with the terms applicable for US Federal Government officers or employees acting as part of their official duties; on a worldwide, perpetual, irrevocable, royalty-free basis to BMJ Publishing Group Ltd ("BMJ") its licensees and where the relevant Journal is co-owned by BMJ to the co-owners of the Journal, to publish the Work in this journal and any other BMJ products and to exploit all rights, as set out in our licence.

The Submitting Author accepts and understands that any supply made under these terms is made by BMJ to the Submitting Author unless you are acting as an employee on behalf of your employer or a postgraduate student of an affiliated institution which is paying any applicable article publishing charge ("APC") for Open Access articles. Where the Submitting Author wishes to make the Work available on an Open Access basis (and intends to pay the relevant APC), the terms of reuse of such Open Access shall be governed by a Creative Commons licence – details of these licences and which Creative Commons licence will apply to this Work are set out in our licence referred to above.

Other than as permitted in any relevant BMJ Author's Self Archiving Policies, I confirm this Work has not been accepted for publication elsewhere, is not being considered for publication elsewhere and does not duplicate material already published. I confirm all authors consent to publication of this Work and authorise the granting of this licence.

For peer review only

Why "one size fits all" is not enough when designing COVID-19 immunity certificates for domestic use: a UK wide cross-sectional online survey

Corina-Elena Niculaescu

Department of Computer Science, Brunel University London, UK, ORCID 0000-0002-8971-0812

Isabel Karen Sassoon

Department of Computer Science, Brunel University London, UK, ORCID 0000-0002-8685-1054

Irma Cecilia Landa-Avila

School of Design and Creative Arts, Loughborough University, UK, ORCID 0000-0001-6107-6736

Ozlem Colak

School of Design and Creative Arts, Loughborough University, UK, ORCID 0000-0003-0813-2561

Gyuchan Thomas Jun

School of Design and Creative Arts, Loughborough University, UK, ORCID 0000-0002-0958-0107

Panagiotis Balatsoukas

School of Design and Creative Arts, Loughborough University, UK, ORCID 0000-0002-2454-0816

For peer review only

Abstract

Objectives: The present study explored public's willingness to use COVID-19 immunity certificates across six different domestic scenarios.

Design: Cross-sectional online survey.

Setting: UK representative survey conducted on the 3rd of August 2021.

Participants: 534 UK residents over 18 years old.

Interventions: Participants replied to the same set of questions.

Primary and secondary outcome measures: The primary outcome measure was *willingness to use* immunity certificates across three different domestic settings (1. Visiting the GP for a non-urgent health issue, 2. Dining in a restaurant, and 3. Attending a performance in a theatre). For each setting two options, one prioritising *convenience* (option A) and the other *privacy* (option B), were offered. Our secondary outcome measures were computed indices from items adapted from the Health Belief Model; Attitudes towards sharing immunity status with service providers; Prior to COVID-19 lifestyle. In addition, we recorded data about respondents' socio-demographic characteristics.

Results: Respondents were more willing to use immunity certificates that prioritised *convenience*, rather than *privacy,* when visiting their GP (92%). However, privacy was more favorable (84%) in the other two settings (dining in a restaurant and going to a theatre) compared to *convenience* (39%). Personal beliefs about COVID-19 and immunity certificates were associated with variations in willingness to use these across all scenarios. No variations were observed across socio-demographics and lifestyle.

Conclusions: The findings of this survey suggest that there is not *one size fits all* solution for designing immunity certificates. Immunity certificates are complex socio-technical systems, any attempt to implement these for domestic use should be tailored to different settings and user needs. The design of certification services requires a more evidence-based approach and further research is needed to understand how different settings, design elements (like *convenience* or *privacy*) and personal beliefs about the pandemic should inform their design.

Keywords: COVID-19, service design, public health, vaccine certificates, immunity certificates, survey

Word count: 4,204

For peer review only

Strengths and limitations of this study

- This study presents unique knowledge about the public's willingness to use immunity certificates for domestic purposes where there is very little published research at the moment.
- The study reports knowledge about the interaction between individual characteristics, domestic settings, and types of immunity certificate design on willingness to use these certificates.
- UK nationally representative sample for age, gender, and ethnic background, but limited to people who have the means and capacity to use digital technologies (survey administered using Prolific.co).
- We present evidence-based recommendations for Public Health services and policy makers about the use of immunity certificates in different domestic settings.
- Since, as to the writing of this paper, COVID-19 certification has not been mandated in the UK, the scenarios used in the survey were hypothetical.

For peer review only

1. Introduction

Since the beginning of the COVID-19 pandemic immunity or vaccine certificates and their adoption by the public for domestic use has sparked a debate. The source for this debate can be attributed to several factors such as the uncertainty around the concept of immunity itself (e.g. what does it really mean to be immune against COVID-19 and how long does this last?)[1–3], the almost antagonist tension between the protection of public health and the respect for human rights or civil liberties (e.g. potential for creating inequality between those who are "immunoprivileged" and those who are "immunodeprived") as well as loss of autonomy[2,4–8], legal challenges in implementing COVID-19 certification across different industries[9,10], risk of fraud and identity theft[5], and fear of digital exclusion[11–13]. Nonetheless, alongside the aforementioned concerns some potential benefits of immunity certificates have been reported such as preserving freedom of movement[14], re-opening the economy[15], reducing risk of infection and social benefits from increased social engagement[11,15].

The debate on the above issues has been reported both among scientists and the public. In a survey of 12,738 scientists from 63 countries, while roughly 22% of the respondents reported concerns around inequality, more than half of them agreed that immunity certificates would be beneficial for the economy and public health[15]. In a different study, textual analysis of Twitter sentiment also showed that in the UK and US vaccine certificates were associated with positive points among the public such as economic recovery, return to normality, safety, return to work or international travel, alongside negative connotations of discrimination[13], surveillance or civil liberties[16]. Similar were the findings reported by another UK nationwide questionnaire survey exploring public attitudes towards vaccine passports[17]. The findings of this survey also showed that people from ethnic minority backgrounds and lower income (less than £20K) would feel unfairly discriminated from the use of vaccine passports.

In the present study we approach immunity certification as a complex socio-technical system. Unlike previous published research we hypothesize that in order to understand what is the best way to design immunity certificates for domestic use we need to investigate the role of different contextual and situational factors, including different types of designs for COVID-19 certification, settings, individual characteristics and their interaction.

The present paper aims to progress the conversation around COVID-19 certification by answering two main research questions. **First, would a design that promotes *convenience* or *privacy* increase willingness to use immunity certificates across three different domestic settings (1. visiting the GP for a non-urgent health issue, 2. Dining in a restaurant, and 3. Attending a performance in a theatre)? Second, what is the role of socio-demographic characteristics, lifestyle, attitudes towards service providers and personal beliefs about COVID-19 on the**

For peer review only - http://bmjopen.bmj.com/site/about/guidelines.xhtml

aforementioned question? To address these two questions, we ran a nationally representative online questionnaire survey in the United Kingdom. Our findings produced unique knowledge about the interaction between individual characteristics, settings of use and types of certificate design on willingness to use immunity certificates. This knowledge can inform current policy on COVID-19 certification and lead to further development of existing solutions for domestic use, such as the UK's NHS COVID Pass[18].

In the present paper the term "immunity certificate" refers to a type of certification (digital or paper) showing that an individual has developed antibodies of SARS-COV-2 either through past infection or after completing a full course of vaccination. We chose to focus on the concepts of *convenience* and *privacy* because both concepts have been reported frequently in the literature as key factors of user experience influencing use and/or adoption of these certificates among the public[12,19–23]. Purposefully, we selected to present *convenience* and *privacy* as two extreme options to understand how willingness to use immunity certificates may be affected across different settings and individual characteristics. This decision was influenced by the findings of our previous studies where research participants perceived the two concepts not as complementary components to the design of services for immunity certificates but as antagonist elements that bring tension and dilemmas[24]. Therefore, in the context of the present study, we hypothesised ***convenience*** as the shift of responsibility for proving an individual's immunity status, from the individual itself, to the service provider whose services the individual wishes to use. For example, when visiting a theatre to watch a performance it is the responsibility of the theatre company to verify a customer's immunity status directly with the NHS without further involvement from the customer. On the other hand, ***privacy*** was defined as people being actively in control about sharing their immunity status and therefore being responsible to decide when, where and with whom their status could be shared[25]. However, this would require additional effort on their behalf including to obtain and share proof of their immunity status with service providers in order to use/access a service (e.g. going to a restaurant). Finally, we selected to focus on the specific three domestic settings (GP appointment for a non-urgent health matter, dining in a restaurant and going to a theatre) because all three represent common, yet distinct (in terms of purpose and social behaviours evoked), types of services that take place indoors.

2. Methods

2.1. Design & Methods

Our analysis was based on a cross-sectional dataset generated from an online questionnaire survey that took place on the 3rd of August 2021. The online questionnaire was created using the *onlinesurveys.ac.uk* platform and administered via *prolific.co.* All the materials related to this

For peer review only - http://bmjopen.bmj.com/site/about/guidelines.xhtml

survey including survey instrument, raw dataset, statistical code, and ethics approval are available on OSF (https://osf.io/jubv6/ DOI:10.17605/OSF.IO/JUBV6).

2.1.1. Sample Design

Respondents were demographically representative of the UK population in terms of gender, age, and ethnicity. Summary statistics for all demographic variables can be found in the Supplementary Material. We excluded 20 participants who failed the attention checks, and one duplicate responder, resulting in a final sample of 534 respondents. All participants were 18 or older and were compensated for their participation in the study with £1.75/person.

2.1.2. Patient and Public Involvement

The research questions and contents of the survey were informed by the findings of a series of studies (composed of focus groups and interviews) which involved a diverse group of research participants including patients, members of the public and service providers from the cultural, sports, hospitality and aviation sectors[24].

2.2. Main variables measure

2.2.1. Willingness to use Immunity Certificates across different scenarios (Primary Outcome)

Each scenario was presented in the form of a short narrative description of a hypothetical use case that combined one of the three settings of interest, as described in section 1, (1. Visiting the GP for a non-urgent health issue; 2. Dining in a restaurant; 3. Attending a performance in a theatre), with one of the following two options: A. Convenience; And B. Privacy. Since the process of using COVID-19 certification for domestic purposes has not been mandated to date in the UK, and there is lots of speculation about how these could become operational in practice, the six scenarios explored hypothetical or future situations balancing imagination/creativity with rigorous reasoning techniques[24,26]. For illustration purposes, Figure 1 presents the six scenarios. In the case of the *convenience* option the service providers in each setting (e.g. restaurant management or theatre company) had the authority to check a customer's COVID-19 immunity status with the NHS, without any additional steps on behalf of the users to prove their status. In the other option (*privacy option*) according to the scenario users needed to generate and share a QR code upon booking an appointment or making a reservation with the service provider to demonstrate their immunity status. In this case, users were actively in control of the process of sharing their immunity status but they had to perform a series of actions to generate/obtain and share the QR code[19,24].

[Figure 1]

For peer review only - http://bmjopen.bmj.com/site/about/guidelines.xhtml

In each of the six scenarios willingness to use immunity certificates was measured by asking respondents to rate how likely they were to use these based on a 5-point Likert Scale (ranging from “Extremely Unlikely” to “Extremely Likely”). Following the data collection, positive to neutral answers, including “Extremely Likely” “Somewhat Likely” and “Neither Likely nor Unlikely” were grouped together under “Yes” describing willingness to use the service. Negative answers including “Extremely Unlikely” and “Somewhat Unlikely” were grouped together under “No” describing reluctance to use the service. We dichotomised the scale this way to make its interpretation easier, and to differentiate between those willing to use the service (neutral to positive answers) and those resistant (negative answers). A similar transformation of 5-Point Likert scale likelihood variables was used in previously published research reporting results from a series of surveys on adherence to test, trace and isolation measures in the UK[27].

2.2.2. Health-Belief Model (Secondary Outcome)

We used a selection of items adapted from the Health Belief Model (HBM)[28] to examine whether certain health beliefs towards vaccination and COVID-19 could influence respondents' willingness to use immunity certificates across the six scenarios. The detailed description of the items, summary statistics and internal reliability measures are presented in Table 1. Each item was rated on a 5-point Likert scale from 1 (“Strongly disagree”) to 5 (“Strongly agree”). First, we measured *COVID-19 Susceptibility* (respondent's perceived susceptibility) using three items adapted from[29] and one item from[30]. Second, we measured *Certificate Severity* (as the perceived severity of not using immunity certificates) using an index of six items. Additional HBM constructs were measured in our survey, but their analysis is beyond the scope of this paper and therefore these are not shown in Table 1.

Table 1 Summary statistics and reliability of HBM measures, and willingness to share immunity status

	**Survey Items**	**Mean**	**Median**	**Std. Dev**	**Min**	**Max**	**Alpha**
COVID-19 Perceived Susceptibility (HBM)	I am at risk of getting COVID-19 (SARS-CoV-2).	3.5243	4	1.1255	1	5	0.7095
	It is likely that I will get COVID-19 (SARS-COV-2).	2.9401	3	1.0122	1	5	
	Individuals in my household are at risk for getting COVID-19 (SARS-COV-2).	3.4438	4	1.1310	1	5	
	I feel knowledgeable about my risk of	4.1255	4	0.7460	1	5	

For peer review only - http://bmjopen.bmj.com/site/about/guidelines.xhtml

	getting COVID-19 (SARS-COV-2).						
Certificate Severity (HBM)	I feel that without this service I won't be able to return to my workplace.	2.4476	2	1.1558	1	5	0.8485
	I feel that without this service my chances of getting a job will be affected.	2.5918	3	1.1631	1	5	
	I feel that without this service I won't be able to book face-to-face appointments with my GP/dentist.	2.8371	3	1.2455	1	5	
	I feel that without this service I won't be able to go to the theatre/movies/sports events.	3.2715	4	1.1636	1	5	
	I feel that without this service I won't be able to travel internationally.	3.912	4	1.1252	1	5	
	I feel that without this service I will not enjoy the same liberties I did before the pandemic.	3.6667	4	1.1692	1	5	
Willingness to Share Immunity Status with Service Providers	Theatre/cinema/galler y	3.2921	4	1.3998	1	5	-
	Pub/restaurant.	3.2228	4	1.4159	1	5	
	GP/dentist	4.47	5	0.9219	1	5	
	Hospitality sector	3.4663	4	1.3717	1	5	
	Sports event	3.3015	4	1.4012	1	5	
	Airport/airline	3.8764	4	1.2538	1	5	

As it is shown in Table 1, *COVID-19 Susceptibility* generated a Cronbach's Alpha of 0.7095 and Certificate Severity a score of 0.8 suggesting good internal consistency[31]. Therefore, we created an index (*Certificate Severity;* and *COVID-19 Susceptibility)* for each of these two constructs by averaging the items within the constructs[19]. Then we used these indices to explore whether there were potential factors influencing willingness to use immunity certificates across the different scenarios.

2.2.3. General attitudes towards sharing Immunity Status with Service Providers (Secondary Outcome)

For peer review only - http://bmjopen.bmj.com/site/about/guidelines.xhtml

Respondents were asked also to rate their willingness to share their immunity status with the following types of service providers: primary care GP/dentist, airport/airline, hospitality (pub/restaurant and hotel), cultural and creative industries (theatres/cinemas/galleries) and sports event organisers (such as football clubs). Responses were recorded using a 5-point Likert scale ranging from 1 (“Strongly disagree”) to 5 (“Strongly agree”). We used these items to examine whether willingness to use immunity certificates measured by our primary outcome was affected by willingness to share immunity status with different service providers in general.

2.2.4. Prior to COVID-19 Lifestyle (Secondary Outcome)

We asked a series of lifestyle questions to determine if respondents’ habits before the COVID-19 outbreak correlated with the willingness to use immunity certificates in the different scenarios. Lifestyle questions recorded the respondents’ perceived frequency of attending or pursuing various activities of immediate interest to the scenarios under investigation including going to the theatre or other cultural venues (like museums and galleries), going to pubs, restaurants, and other dining venues, or visiting health care settings. Other lifestyle questions collected data about their frequency of travelling abroad, and booking accommodation when travelling abroad, Responses were captured using a 4-point Likert scale ranging from 1 (“Never”) to 4 (“Very often”).

2.3. Statistical Analysis

To address the first research question, this survey design enabled us to analyse the responses collected for the primary outcome measure (i.e., willingness to use immunity certificates across different scenarios) as a repeated measures 2*3 factorial design with two fixed effects (the *convenience/privacy options* and the setting) and with a random effect of the responder. The dependent variable for this analysis was the willingness to use immunity certificates. Each responder had six willingness to use responses each corresponding to a different scenario which was the result of the combination of *convenience* versus *privacy* options and settings[32].

To analyse whether there was a significant difference in the likelihood of using option A (*convenience*) and B (*privacy*) in each of the three settings we employed a Generalised Linear Mixed Effects Model (GLMM)[32,33]. We fit the GLMM model which incorporates both fixed effects parameters (*convenience/privacy* and setting) and random effects in a linear predictor, via maximum likelihood.

Finally, to address our second research question, we employed an exploratory analysis through graphical representations of the relationships between the dependent variables (primary outcome measure) and the other secondary outcome measures. To display relationships between the primary outcome measure and the HBM indices we used box plot graphs, and for the rest of the variables we present mosaic plots. The statistical analysis was performed in STATA[34] and R[35].

For peer review only - http://bmjopen.bmj.com/site/about/guidelines.xhtml

2.4. Power calculation

The sample size was chosen pragmatically based on several different approaches, obtaining a minimum sample size between 271 and 1,067 participants, depending on the assumptions. This sample size results in a 99% power in the GLMM model used in our statistical analysis of the first research question.

3. Results

3.1. Does a person's willingness to use immunity certificates vary across the six scenarios?

Figure 2 illustrates the distribution of responses across the six scenarios while Table 2 the proportion of respondents who would be willing to use the certificates across these scenarios. The data show that the majority of respondents (92%) were more willing to use immunity certificates that prioritised *convenience* when visiting their GP (Scenario 1A). However, *convenience* was less favorable in the other two settings with only 38% and 39% of respondents willing to use the certificates for dinning indoors (Scenario 2A) and going to the theatre (Scenario 3A) respectively.

[Figure 2]

For peer review only

Table 2 Acceptance of different service designs and settings

Response	NO	YES
Scenario 1A: Visiting the GP/Convenience option	44	490 (92%)
Scenario 1B: Visiting the GP /Privacy option	129	405 (76%)
Scenario 2A: Dining in a Restaurant/Convenience option	329	205 (38%)
Scenario 2B: Dining in a Restaurant/Privacy option	85	449 (84%)
Scenario 3A: Attending a Performance in the Theatre/Convenience option	324	210 (39%)
Scenario 3B: Attending a Performance in the Theatre/Privacy option	89	445 (83%)

Table 3 Summary of the coefficients of the Generalized linear mixed model fit

Fixed Effects	Estimate	Std. Error	z value	Pr (>|z|)
(Intercept)	4.1636	0.2646	15.7300	<2e-16 ***
Dining in a Restaurant Setting	-5.0121	0.2837	-17.6700	<2e-16 ***
Attending a Performance in the Theatre Setting	-4.9398	0.2822	-17.5000	<2e-16 ***
Privacy Option	-2.0736	0.2504	-8.2800	<2e-16 ***

For peer review only - http://bmjopen.bmj.com/site/about/guidelines.xhtml

Dining in a Restaurant Setting Setting*Privacy Option	5.8747	0.3595	16.3400	<2e-16 ***
Attending a Performance in the Theatre Setting*Privacy Option	5.7127	0.3557	16.0600	<2e-16 ***

Note: Table 3 contains the coefficients in log-odds form of the GLMM model and their significance. The estimates of the fixed effects are conditional on the random effects. The estimated effects have a binary outcome with a logit link; hence the raw estimates are on the log-odds scale. The intercept refers to the log-odds for willingness to use immunity certificates in Scenario 1A (visiting the GP/Convenience option). A positive value for log-odds estimates respondents being likely to be willing to use the certificate in that setting and option. The coefficients on the log-odds scale are additive.
*Significance codes 0 '***'; 0.001 '**'; 0.01;'*'; 0.05 '.'; 0.1 ' '*

To determine whether these differences in willingness to use were statistically significant we applied the GLMM model, including fixed and random effects outlined in Section 2.3. Table 3 includes a subset of the R output for the GLMM analysis (logistic regression with mixed fixed and random effects). The estimated coefficient for the intercept 4.1636, is the log odds for scenario 1A (choosing *convenience* option for visiting the GP for a non-urgent health issue). The estimate of -5.0121 for dinning in a restaurant means that this setting is associated with a 5.0121 decrease in the log-odds of positive response ("Yes") compared to negative response ("No"). The log-odds for scenario 2B (choosing *privacy* for dining in a restaurant) is the sum of the intercept (4.1636), the indoor dining setting (-5.0121), the *privacy* option (-2.0736), and their interaction (5.8747), resulting in a log-odds of 2.9526. This points to a higher likelihood of willingness to use immunity certificates for scenario 2B (choosing *privacy* for dining in a restaurant) than for scenario 2A (choosing *convenience* for dining in a restaurant).

The likelihood of using the certificate when going a restaurant or to the theatre was significantly lower than visiting the GP with a statistically significant p-value<0.001 when considering the scenarios irrespective of the option. Also, the likelihood of using the certificate was significantly lower for *privacy* than *convenience* when visiting the GP (p-value<0.001). However, when considering the *privacy option* in the restaurant or theatre setting, this likelihood of using the certificate is higher and statistically significant (p-value<0.001).

3.2. What is the role of personal beliefs about COVID-19 (certificate severity and COVID-19 susceptibility), attitudes towards service providers, lifestyle, and socio-demographic characteristics on the willingness to use immunity certificates?

To address our second research question, we graphically explored the primary outcome variables against all other secondary outcome variables, but for brevity we only present here the key results.

- *Certificate severity*. Lower values for *Certificate Severity* suggest that respondents did not perceive immunity certificates as necessary (Figure 3A). We observed lower perceived

For peer review only - http://bmjopen.bmj.com/site/about/guidelines.xhtml

Certificate severity among those who were not willing to use immunity certificates. This finding was observed across all settings and for both options (*convenience* and *privacy*).

[Figure 3]

- *COVID-19 susceptibility*. The median of *perceived susceptibility* is consistently higher for responses indicating willingness to use immunity certificates across all scenarios even when these prioritised *convenience* (Figure 3B). This means that respondents who perceived themselves as being more susceptible to COVID-19 were more willing to use immunity certificates for both options. Also, the median values show that this group of respondents would be more willing to use an option that prioritised *convenience* when going to a restaurant or the theatre rather than when visiting their GP for a non-urgent health matter. This finding suggests that people who perceived themselves to be at high risk from COVID-19 were more willing to trade-off *privacy* for *convenience* for specific settings.

- *General attitudes towards sharing immunity status with service providers*. Respondents who were comfortable sharing their COVID-19 immunity status with service providers from the cultural and creative industries (e.g. theatres, cinemas or galleries) were more willing to use immunity certificates across all three settings (Figure 4). Also, this group of respondents was more likely to choose options prioritising *convenience*. On the contrary respondents who did not like sharing their immunity status with service providers from the creative and cultural industries were less willing to use immunity certificates across all scenarios even when this prioritised *privacy*. Similar patterns with those exhibited in the case of cultural and creative industries were observed in the case of the other types of service providers, including hospitality, airlines/airports, sports events, and pubs/restaurants.

[Figure 4]

- *The role of socio-demographics and lifestyle characteristics.* Our exploration of the relationship between willingness to use the immunity certificates across the six scenarios did not point to any significant differences across socio-demographics or lifestyle. Specifically, we observed no variation in willingness to use immunity certificates across the different scenarios by gender, age, and ethnicity. Also, willingness to use immunity certificates did not vary by other socio-demographics such as disability, living arrangements and living in a rural versus urban area. In addition, lifestyle characteristics

For peer review only - http://bmjopen.bmj.com/site/about/guidelines.xhtml

were not associated with variations in willingness to use immunity certificates either. Lastly, there was no variation in willingness to use the service based on mental wellbeing and net income now as compared to before the outbreak, suggesting that willingness to use immunity certificates does not stream from feelings of hopelessness.

4. Discussion

Practical implication for policy making

As of the writing of this paper (September 2021), the use of immunity certificates for domestic purposes in the UK was not mandatory. Also, there was still lack of knowledge or guidance about what is the best way to design immunity certificates for use in different settings. Therefore, the present survey contributes unique knowledge that different stakeholders (the NHS, the UK government and the Scientific Advisory Group for Emergencies, businesses, and the public) should take into account when considering the use of immunity certificates for domestic purposes. Some key implications of this study for policy making are the following:

- *Use of certification in primary care settings*. Participants' responses showed high willingness to use immunity certificates when visiting their GP for a non-urgent health matter compared to the other settings (dinning in a restaurant and going to the theatre). In this case the majority of responses were positive for both options (*privacy* and *convenience*). We argue that the implementation of immunity certificates for non-urgent healthcare matters could play a role in increasing the sense of safety as well as reducing waiting times for face-to-face appointments especially when the option that prioritises *convenience* is selected to confirm patients' immunity status.

  However, as of today, in the UK, the use of any type of immunity certificate in healthcare settings is not recommended. Currently consultations with a GP can be either remote (videoconferencing or via telephone) or face-to-face. The transition to pre-pandemic appointments is not fully completed, and the risks of attending face-to-face appointments are still present. For instance, dental procedures can increase the risk of SARS-CoV-2 transmission due to tools that produce aerosols[36,37], not to mention the issue of contracting the virus in a hospital setting[38,39]. As such patients and health care professionals are presented with the choice between remote appointments for non-urgent matters or the risk of infection in a face-to-face setting. While to a certain extend there is some evidence that remote consultations have been accepted well by the patients[40], several challenges have been reported too, especially in the case of people who suffer from pre-existing chronic conditions or who they may not feel comfortable with the use of technology[40–42]. For those patients, implementation of immunity certificates for face-

For peer review only - http://bmjopen.bmj.com/site/about/guidelines.xhtml

to-face appointments would be important in improving their sense of safety. In addition, our findings showed that as opposed to the other domestic settings, in the case of visiting a GP for a non-urgent matter, respondents were more willing to use immunity certificates that prioritised *convenience*. This means that access to primary care services should be seamless without a need for patients to show proof of their immunity status in a digital or physical format. Instead, this proof could be verified by the GP practice upon booking an appointment (over the phone or electronically). This would result in zero checks at the reception, shorter queues upon arrival and better experience for the patients.

- *Use of certification for social indoor activities.* When it comes to leisure activities (such as dining in a restaurant or going to a theatre) respondents were less willing to use immunity certificates, and if they did most of them would value the *privacy* option over *convenience*. One key implication of this finding when designing certification services is to make clear to the public how data *privacy* and protection applies to this context. The findings of our previous research[24] suggest that the public is not convinced about whether or not their data is shared when using these certificates, thus making them more sceptical to use these in settings where they feel that they are less secure or they do not trust. This happens despite the fact that in the UK verification of someone's immunity status using the paper or digital certification format (via the NHS app[18]) does not involve sharing of any personal information with the service provider (for example, the theatre company or the restaurant)[43]. For the use and uptake of such certificates to be successful the public and businesses should be educated, and nation-wide public health campaigns should promote this shared understanding explaining the extent to which personal data, if any, is shared upon verification of someone's immunity status.
- *Need to build empathy and understand public's views about COVID-19 when designing certification services*. While lots of attention when designing different forms of COVID-19 certification was placed on issues surrounding their accessibility[13,44–46], less effort has been put in place to harness public's beliefs around COVID-19 and COVID-19 certification and fed these into the design of services for immunity certificates. The implementation of immunity certificates should be accompanied by a series of health promotion strategies tailored to target the needs of people with different beliefs, knowledge and understanding about COVID-19, and ultimately change their behaviour. The present survey showed that traditional demographic information and lifestyle does not influence user willingness to use immunity certificates across the six scenarios. However, our findings also showed that perceived risk of falling severely ill from COVID-19 (*COVID-19 susceptibility*) and perceived severity from not using immunity certificates (*Certificate severity*) can influence public's willingness to use immunity certificates.

For peer review only - http://bmjopen.bmj.com/site/about/guidelines.xhtml

Limitations

One of the limitations of our study is that participants were recruited using the online survey platform Prolific. Since surveys administered via this platform are completed online (mobile, PC, tablet etc.) our sample is comprised of people who had the means and capacity to use digital technologies. Other studies investigating people's perceptions of immunity certificates[13,19,47] or COVID-19 vaccine intentions[27,48–50] found some differences based on gender and ethnicity, which we did not find. This can be explained by the fact that unlike the other cited studies, our survey was focused on six specific hypothetical scenarios of using immunity certificates. Another possible explanation can be attributed to the timing of this survey. At the time our survey took place immunity certificates were being used for international travel, hence access, awareness and familiarity with such services were higher than in previously published studies. Finally, from a methodological point of view the mixed effects model we used assumed that the random effect subsumes the possible effects of gender and ethnicity, as we are looking at responses of the same person. Therefore, both the scope and timing of the present study as well as the mixed effects model used differed from other surveys in this context.

5. Conclusions

The findings of this survey suggest that there is not *one size fits all* solution for designing immunity certificates. Immunity certificates should be studied as complex socio-technical services rather than merely products that one can simply download and use. Any attempt to implement such certificates for domestic use should be tailored to different settings and user needs. While some implications of our findings for policy making were discussed, the design of certification services requires a more evidence-based approach and further research is needed to examine willingness to use immunity certificates across the present three, and possibly other, domestic settings. Also, while in the present survey we chose to explore two options for designing immunity certificates (one prioritising *convenience* and the other *privacy*), our previous work[51] has shown that there are still more attributes that may influence use in this context and future research should focus on.

For peer review only - http://bmjopen.bmj.com/site/about/guidelines.xhtml

Footnotes

Contributors: The questionnaire survey was conceptualised by CN, IS and PB, with the input of TG, CLA, and OC. CEN and PB completed the data collection. CN and IS conducted the statistical analysis. All authors contributed and approved the final manuscript.

Funding: IMMUNE or Immunity Passport Service Design is a nine-month project funded by the AHRC/UKRI COVID-19 Rapid Response (Ref. AH/W000288/1).

Ethics statements: Ethics approval was obtained from the College of Engineering, Design and Physical Sciences Research Ethics Committee at Brunel University London (Ref. 31705-A-Jul/2021- 33586-1) on the 29th of July 2021.Informed consent was obtained from all respondents prior to the beginning of the survey. Respondents were allowed to withdraw from the survey at any time.

Data availability statement: The data is available in a public, open access repository. All statistical code, dataset, survey item and ethical approval are available on OSF (https://osf.io/jubv6/), DOI 10.17605/OSF.IO/JUBV6.

Competing interests: None declared.

For peer review only

References

1 Wilf-Miron R, Myers V, Saban M. Incentivizing Vaccination Uptake: The ‘green Pass’ Proposal in Israel. *JAMA - J Am Med Assoc* 2021;**325**:1503–4. doi:10.1001/jama.2021.4300

2 Larremore DB, Bubar KM, Grad YH. Implications of Test Characteristics and Population Seroprevalence on ‘Immune Passport’ Strategies. *Clin Infect Dis* 2021;**72**:e412–4. doi:10.1093/cid/ciaa1019

3 Liew CH, Flaherty GT. Immunity passports to travel during the COVID-19 pandemic: controversies and public health risks. *J Public Health (Oxf)* 2021;**43**:e135–6. doi:10.1093/pubmed/fdaa125

4 Voo TC, Reis AA, Thomé B, *et al.* Immunity certification for COVID-19: Ethical considerations. *Bull World Health Organ* 2021;**99**:155–61. doi:10.2471/BLT.20.280701

5 Liz J. COVID-19, immunoprivilege and structural inequalities. *Hist Philos Life Sci* 2021;**43**:1–6. doi:10.1007/s40656-020-00356-5

6 Bramstedt KA. Antibodies as Currency: COVID-19’s Golden Passport. *J Bioeth Inq* Published Online First: 2020. doi:10.1007/s11673-020-09996-5

7 Brown RCH, Savulescu J, Williams B, *et al.* Passport to freedom? Immunity passports for COVID-19. *J Med Ethics* 2020;**46**:652–9. doi:10.1136/medethics-2020-106365

8 Kosciejew MRH. COVID-19 immunity (or vaccine) passports: a documentary overview and analysis of regimes of health verification within the coronavirus pandemic. *J Doc* 2021;**ahead-of-p**. doi:10.1108/JD-04-2021-0079

9 Voo TC, Clapham H, Tam CC. Ethical implementation of immunity passports during the COVID-19 pandemic. *J Infect Dis* 2020;**222**:715–8. doi:10.1093/infdis/jiaa352

10 Phelan AL. COVID-19 immunity passports and vaccination certificates: scientific, equitable, and legal challenges. *Lancet* 2020;**395**:1595–8. doi:10.1016/S0140-6736(20)31034-5

11 Häkli J. COVID-19 Certificates as a New Form of Mobility Control. *Eur J Risk Regul* 2021;:1–8. doi:10.1017/err.2021.21

12 Wilford SH, McBride N, Brooks L, *et al.* The Digital Network of Networks: Regulatory Risk and Policy Challenges of Vaccine Passports. *Eur J Risk Regul* 2021;:1–11. doi:10.1017/ERR.2021.35

For peer review only - http://bmjopen.bmj.com/site/about/guidelines.xhtml

13 Ada Lovelace Institute. The data divide: Public attitudes to tackling social and health inequalitites in the COVID-19 pandemic and beyond. 2021.

14 Persad G, Emanuel EJ. The Ethics of COVID-19 Immunity-Based Licenses ("Immunity Passports"). *JAMA* 2020;**323**:2241–2. doi:10.1001/jama.2020.8102

15 Aranzales I, Chan HF, Eichenberger R, *et al.* Scientists have favorable opinions on immunity certificates but raise concerns regarding fairness and inequality. *Sci Rep* 2021;**11**:14016. doi:10.1038/s41598-021-93148-1

16 Uk W, Think R, Passport AV. Have Vaccine Passport Will Travel: What UK Residents Think About Vaccine Passport. 2021.

17 Ada Lovelace Institute. Public attitudes towards vaccine passports. 2021.https://www.adalovelaceinstitute.org/survey/public-attitudes-towards-vaccine-passports/ (accessed 10 Sep 2021).

18 Department of Health and Social Care. NHS COVID Pass. 2021.https://www.gov.uk/guidance/nhs-covid-pass (accessed 27 Sep 2021).

19 Lewandowsky S, Dennis S, Perfors A, *et al.* Public acceptance of privacy-encroaching policies to address the COVID-19 pandemic in the United Kingdom. *PLoS One* 2021;**16**:1–23. doi:10.1371/journal.pone.0245740

20 Hasan HR, Salah K, Jayaraman R, *et al.* Blockchain-Based Solution for COVID-19 Digital Medical Passports and Immunity Certificates. *IEEE Access* 2020;**8**:222093–108. doi:10.1109/ACCESS.2020.3043350

21 Ricci L, Di Francesco Maesa D, Favenza A, *et al.* Blockchains for covid-19 contact tracing and vaccine support: A systematic review. *IEEE Access* 2021;**9**:37936–50. doi:10.1109/ACCESS.2021.3063152

22 Gstrein OJ. The EU Digital COVID Certificate: A Preliminary Data Protection Impact Assessment. *Eur J Risk Regul* 2021;:1–12. doi:10.1017/err.2021.29

23 Papadaki M, Karamitsos I, Themistocleous M. ViewpointCovid-19 digital test certificates and blockchain. *J Enterp Inf Manag* 2021;**34**:993–1003. doi:10.1108/JEIM-07-2021-554

24 Landa-Avila IC, Thomas Gyuchan J, Sassoon I, *et al.* COVID-19 Immunity certificates as complex systems: Applying systems approaches to explore needs, risks, and unintended consequences. In: *Relating Systems Thinking & Design Symposium RSD10*. RSD10 2021.

25 Wolford B. A guide to GDPR data privacy requirements. GDPR EU. https://gdpr.eu/data-privacy/

For peer review only - http://bmjopen.bmj.com/site/about/guidelines.xhtml

26 Ramirez R, Mukherjee M, Vezzoli S, *et al.* Scenarios as a scholarly methodology to produce ‘interesting research’. *Futures* 2015;**71**:70–87. doi:10.1016/j.futures.2015.06.006

27 Smith LE, Potts HWW, Amlôt R, *et al.* Adherence to the test, trace, and isolate system in the UK: results from 37 nationally representative surveys. *bmj* 2021;**372**.

28 Champion VL, Skinner CS. The health belief model. In: *Health behavior and health education: Theory, research, and practice, 4th ed.* San Francisco, CA, US: : Jossey-Bass 2008. 45–65.

29 Coe AB, Elliott MH, Gatewood SBS, *et al.* Perceptions and predictors of intention to receive the COVID-19 vaccine. *Res Soc Adm Pharm* 2021;:1–7. doi:10.1016/j.sapharm.2021.04.023

30 Chu H, Liu S. Integrating health behavior theories to predict American’s intention to receive a COVID-19 vaccine. *Patient Educ Couns* Published Online First: 2021. doi:10.1016/j.pec.2021.02.031

31 Cortina JM. What is coefficient alpha? An examination of theory and applications. *J Appl Psychol* 1993;**78**:98–104. doi:10.1037/0021-9010.78.1.98

32 Agresti A. *Categorical data analysis*. Vol. 482. John Wiley & Sons. 2003.

33 Bates D, Maechler M, Bolker B, *et al.* Mixed-Effects Models Using lme4. *J Stat Softw* 2015;**67**:1–48. doi:10.18637/jss.v067.i01

34 StataCorp. Stata Statistical Software: Release 17. 2021.https://www.stata.com/

35 R Core Team. R: A language and environment for statistical computing. 2021.https://www.r-project.org/

36 Epstein JB, Chow K, Mathias R. Dental procedure aerosols and COVID-19. *Lancet Infect Dis* 2021;**21**:e73. doi:10.1016/S1473-3099(20)30636-8

37 Ge Z yu, Yang L ming, Xia J jia, *et al.* Possible aerosol transmission of COVID-19 and special precautions in dentistry. *J Zhejiang Univ Sci B* 2020;**21**:361–8. doi:10.1631/jzus.B2010010

38 Duncan C. Up to 8,700 patients died after catching Covid in English hospitals, figures show. Indep. 2021.https://www.independent.co.uk/news/health/covid-deaths-in-hospitals-b1853184.html

39 Oliver D. David Oliver: Deaths from hospital acquired covid are everyone’s problem. *BMJ* 2021;**373**:372–3. doi:10.1136/bmj.n1492

For peer review only - http://bmjopen.bmj.com/site/about/guidelines.xhtml

40 Nijhof D, Ingram A, Ochieng R, *et al.* Examining GP online consultation in a primary care setting in east midlands , UK. 2021;:1–10.

41 Quinn-Scoggins HD, Cannings-John R, Moriarty Y, *et al.* Cancer symptom experience and help-seeking behaviour during the COVID-19 pandemic in the UK: A cross-sectional population survey. *BMJ Open* 2021;**11**:1–15. doi:10.1136/bmjopen-2021-053095

42 Hearn JH, Rohn EJ, Monden K. Isolated and anxious: A qualitative exploration of the impact of the COVID-19 pandemic on individuals living with spinal cord injury in the UK. *J Spinal Cord Med* 2021;**0**:1–9. doi:10.1080/10790268.2021.1949562

43 Balatsoukas P, Jun GT, Sassoon I. Vaccine passports: what businesses need to know – and why they should have more say. 2021.https://theconversation.com/vaccine-passports-what-businesses-need-to-know-and-why-they-should-have-more-say-164606?utm_source=twitter&utm_medium=bylinetwitterbutton

44 Ganty S. The Veil of the COVID-19 Vaccination Certificates: Ignorance of Poverty, Injustice towards the Poor. *Eur J Risk Regul* 2021;:1–12. doi:10.1017/err.2021.23

45 Osama T, Razai MS, Majeed A. Covid-19 vaccine passports: access, equity, and ethics. *BMJ* 2021;**373**:n861. doi:10.1136/bmj.n861

46 The Royal Society. Twelve criteria for the development and use of COVID-19 vaccine passports. 2021.

47 Hall MA, Studdert DM, Favorito L, *et al.* U.S. Public Views about COVID-19 'Immunity Passports'. *medRxiv* Published Online First: 2021. doi:10.1101/2021.01.26.21250184

48 Guidry JPD, Laestadius LI, Vraga EK, *et al.* Willingness to get the COVID-19 vaccine with and without emergency use authorization. *Am J Infect Control* 2021;**49**:137–42. doi:10.1016/j.ajic.2020.11.018

49 Paul E, Steptoe A, Fancourt D. Attitudes towards vaccines and intention to vaccinate against COVID-19: Implications for public health communications. *Lancet Reg Heal - Eur* 2021;**1**:100012. doi:10.1016/j.lanepe.2020.100012

50 Blundell R, Costa Dias M, Joyce R, *et al.* COVID-19 and Inequalities*. *Fisc Stud* 2020;**41**:291–319. doi:10.1111/1475-5890.12232

51 Landa-Avila C, Colak O, Niculaescu C, *et al.* Mapping COVID-19 Health Certificates as a Complex System (version 1.1). 2021. doi:10.17028/rd.lboro.14933391.v1

For peer review only

Figure Legends

Figure 1 Description of the six scenarios (the number represents one of the three settings while the letter the design option, convenience or privacy)

Figure 2 Distribution of number of responses (N) across settings (visiting the GP, dining in a restaurant, attending a performance in the theatre) and design options (convenience/privacy).

Figure 3. **A.** Index of Certificate Severity (perceived severity of not using immunity certificates) and **B**. Index of COVID-19 Susceptibility across settings and design options (convenience/privacy).

Figure 4 Willingness to Share Immunity Status with Service Providers (Theatre/Cinema/Gallery) by across settings and design options (convenience/privacy).

For peer review only

For peer review only - http://bmjopen.bmj.com/site/about/guidelines.xhtml

For peer review only

For peer review only - http://bmjopen.bmj.com/site/about/guidelines.xhtml

Scenario 1 (Visiting the GP for a non-urgent health matter)
You want to book a face-to-face appointment with your GP for a non-urgent matter. A non-urgent matter is one that does not warrant using 111 or 999 NHS services. In order to ensure patient and medical personnel safety, your GP practice has health and safety measures in place requiring patients to prove their COVID-19 (SARS-CoV-2) immunity status when booking a face-to-face appointment. Proof of your immunity status will determine the location and time of the appointment. Now, let's consider two different ways in which this service could be implemented:

> **Option 1A, Convenience:** Your GP practice has the authority to check your immunity status upon booking the appointment by checking the information in your NHS patient electronic health record (you'll have to share your NHS number). There are no additional steps on your behalf.

> **Option 1B, Privacy:** Your GP practice is not authorised to check your immunity status by accessing your NHS electronic health record. Instead, you would need either extract a QR code from your smartphone's NHS app and share it with your GP practice before confirming your booking or presenting a formal paper certificate proving your status.

Scenario 2 (Dining in a restaurant)
You want to go out for a meal at a restaurant, which only has indoor tables. In order to ensure the safety of the staff and the other diners, the restaurant has health and safety measures in place requiring customers to prove their COVID-19 (SARS-CoV-2) immunity status when making a reservation. Proof of your immunity status will enable you to dine at this restaurant. If you choose not to share your status, you would not be able to dine at this restaurant or book a table. Now, let's consider two different ways in which this service could be implemented:

> **Option 2A, Convenience:** The restaurant has the authority to check your status with the NHS upon booking a table or at least 48 hours prior. You would share your NHS number with the restaurant to enable them to check your immunity status. You would then be able to dine at the restaurant at your booked time without any extra steps on your behalf.

> **Option 2B, Privacy:** The restaurant has no authority to check your immunity status with the NHS on your behalf. Instead, you would need either to extract a QR code from your smartphone's NHS app and share it with the restaurant or present a formal paper certificate upon confirming your reservation.

Scenario 3 (Attending a performance in the theatre)
You want to attend a play at your local theatre, this is an indoor event. In order to ensure the safety of the audience, actors and the other staff, the theatre has health and safety measures in place requiring customers to prove their COVID-19 (SARS-CoV-2) immunity status when booking a ticket for a play. Proof of your immunity status will enable you to attend this play. If you choose not to share your status, you would not be able to attend this indoor event on the selected day. Now, let's consider two different ways in which this service could be implemented:

> **Option 3A, Convenience:** The theatre company has the authority to check your status with the NHS upon booking a ticket to a play or at least 48 hours prior. You would share your NHS number with the theatre company to enable them to check your immunity status. You would then be able to attend the play without any extra steps on your behalf.

> **Option 3B, Privacy:** The theatre company has no authority to check your immunity status with the NHS on your behalf. Instead, you would need either to extract a QR code from your smartphone's NHS app and share it with the theatre company or present a formal paper certificate upon confirming your booking or on the day of the play.

For peer review only

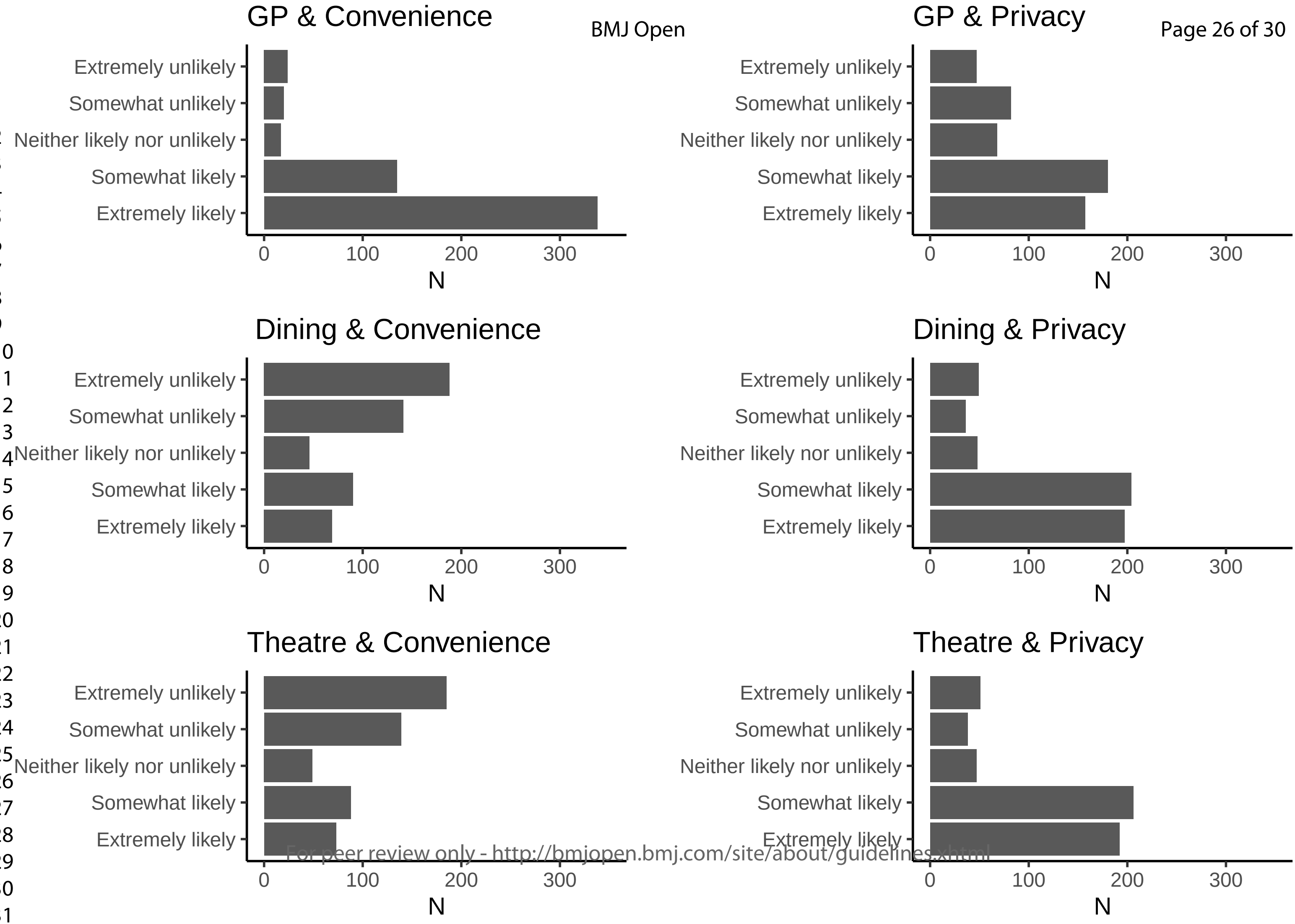

For peer review only - http://bmjopen.bmj.com/site/about/guidelines.xhtml

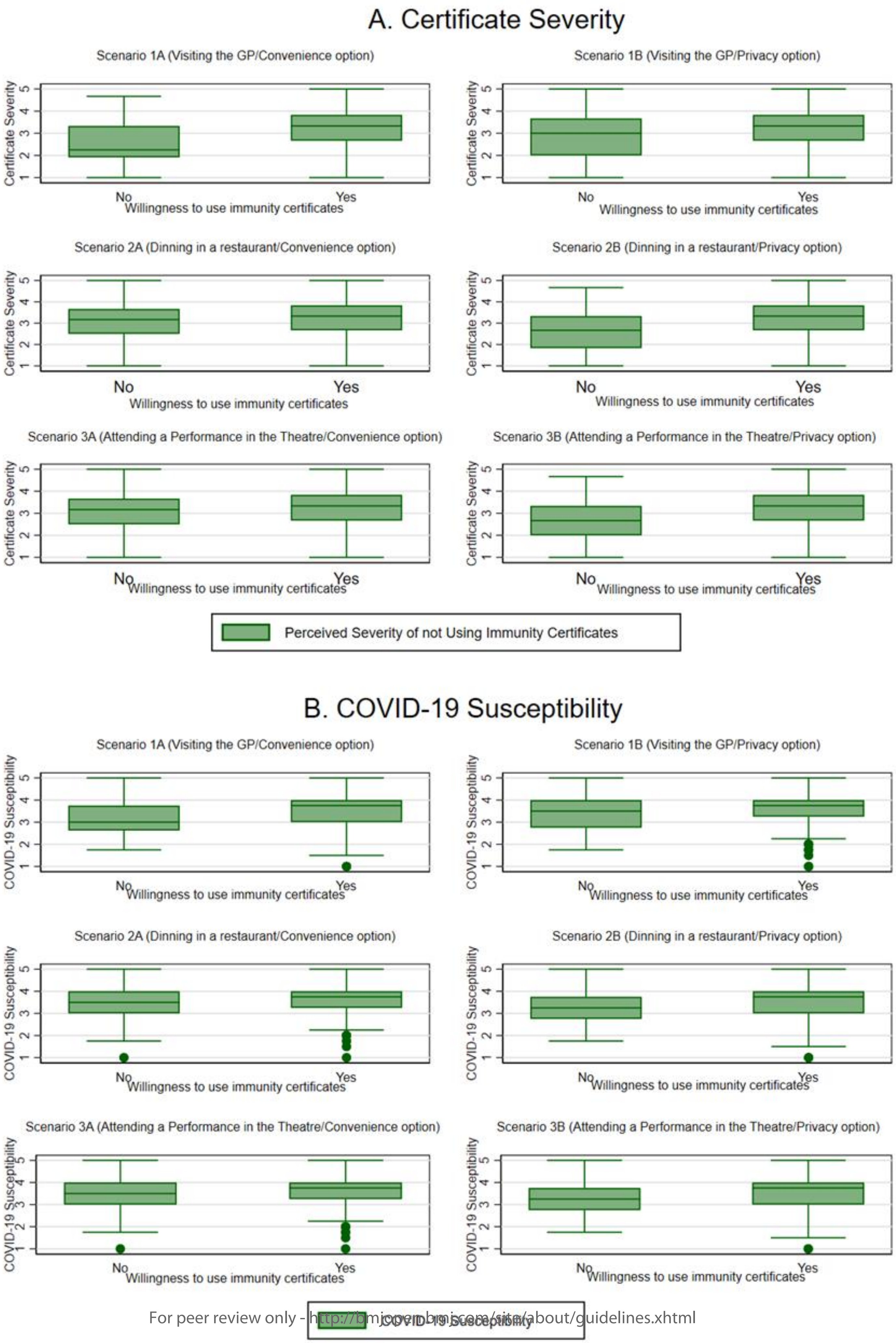
A. Certificate Severity
Scenario 1A (Visiting the GP/Convenience option)
Scenario 1B (Visiting the GP/Privacy option)
Scenario 2A (Dinning in a restaurant/Convenience option)
Scenario 2B (Dinning in a restaurant/Privacy option)
Scenario 3A (Attending a Performance in the Theatre/Convenience option)
Scenario 3B (Attending a Performance in the Theatre/Privacy option)
Certificate Severity
Willingness to use immunity certificates
No
Yes
Perceived Severity of not Using Immunity Certificates
B. COVID-19 Susceptibility
Scenario 1A (Visiting the GP/Convenience option)
Scenario 1B (Visiting the GP/Privacy option)
Scenario 2A (Dinning in a restaurant/Convenience option)
Scenario 2B (Dinning in a restaurant/Privacy option)
Scenario 3A (Attending a Performance in the Theatre/Convenience option)
Scenario 3B (Attending a Performance in the Theatre/Privacy option)
COVID-19 Susceptibility
Willingness to use immunity certificates
No
Yes
COVID-19 Susceptibility

For peer review only

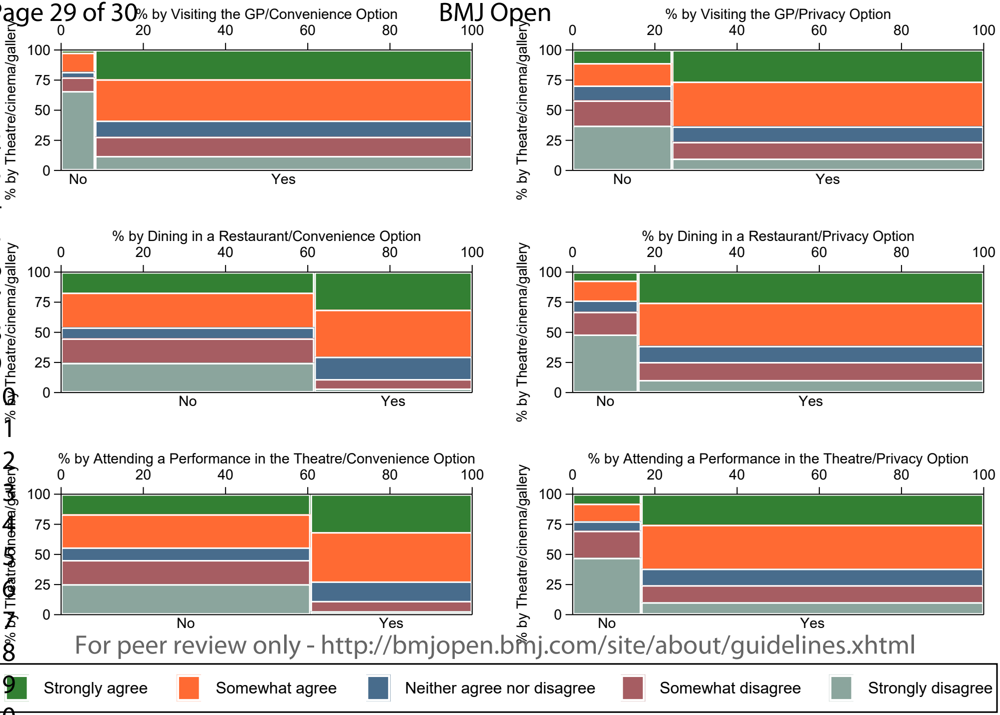

For peer review only - http://bmjopen.bmj.com/site/about/guidelines.xhtml

Supplementary Materials

Supplementary Table 1 Demographic characteristics of sample

		Freq.	Percent	Cum.
**Gender**	Female	277	51.87%	51.87%
	Male	254	47.57%	99.44%
	Self-define	1	0.19%	100%
	Prefer not to say	2	0.37%	99.81%
**Age**	18 – 23	77	14.42%	14.42%
	24 – 29	51	9.55%	23.97%
	30 – 39	95	17.79%	41.76%
	40 – 49	87	16.29%	58.05%
	50 – 59	95	17.79%	75.84%
	60 – 69	109	20.41%	96.25%
	70 or older	20	3.75%	100%
**Ethnicity**	Asian	34	6.37%	6.37%
	Black	20	3.75%	10.11%
	Hispanic/Latino	3	0.56%	10.67%
	Mixed	15	2.81%	13.48%
	Other	8	1.50%	14.98%
	South Asian	12	2.25%	17.23%
	White	442	82.77%	100%
**Region**	East Midlands	42	7.87%	7.87%
	East of England	35	6.55%	14.42%
	London	81	15.17%	29.59%
	Northeast	32	5.99%	35.58%
	Northern Ireland	11	2.06%	37.64%
	Northwest England	58	10.86%	48.50%
	Scotland	37	6.93%	55.43%
	South-East England	87	16.29%	71.72%
	Southwest of England	43	8.05%	79.78%
	Wales	19	3.56%	83.33%
	West Midlands	45	8.43%	91.76%
	Yorkshire and the Humber	44	8.24%	100%
**Area**	Rural	166	31.09%	31.09%
	Urban	368	68.91%	100%
**Accommodation**	Living alone	87	16.29%	16.29%
	Living in shared accommodation	54	10.11%	26.40%
	Living with other family members	382	71.54%	97.94%
	Other	11	2.06%	100%
**Employment**	Employed/Self-employed	340	63.67%	63.67%
	Retired	97	18.16%	81.84%
	Unemployed	97	18.16%	100%
**Education**	A level (or equivalent)	130	24.34%	24.34%
	GCSE (or equivalent)	80	14.98%	39.33%
	Postgraduate degree	95	17.79%	57.12%
	Undergraduate degree	175	32.77%	89.89%
	Vocational	54	10.11%	100%
**Disability**	No	467	87.45%	87.45%
	Prefer not to say	6	1.12%	88.58%
	Yes	61	11.42%	100%
All		*534*	100%	

For peer review only

For peer review only

BMJ Open

Why "one size fits all" is not enough when designing COVID-19 immunity certificates for domestic use: a UK wide cross-sectional online survey

Journal:	*BMJ Open*
Manuscript ID	bmjopen-2021-058317.R1
Article Type:	Original research
Date Submitted by the Author:	09-Mar-2022
Complete List of Authors:	Niculaescu, Corina; Brunel University London, Department of Computer Science<br>Sassoon, Isabel; Brunel University London, Department of Computer Science<br>Landa-Avila, Irma; Loughborough University<br>Colak, Ozlem; Loughborough University<br>Jun, Gyuchan; Loughborough University, Loughborough Design School<br>Balatsoukas, Panagiotis; Loughborough University
**Primary Subject Heading**:	Health informatics
Secondary Subject Heading:	Public health
Keywords:	Public health < INFECTIOUS DISEASES, COVID-19, PUBLIC HEALTH, Health informatics < BIOTECHNOLOGY & BIOINFORMATICS

SCHOLARONE™
Manuscripts

I, the Submitting Author has the right to grant and does grant on behalf of all authors of the Work (as defined in the below author licence), an exclusive licence and/or a non-exclusive licence for contributions from authors who are: i) UK Crown employees; ii) where BMJ has agreed a CC-BY licence shall apply, and/or iii) in accordance with the terms applicable for US Federal Government officers or employees acting as part of their official duties; on a worldwide, perpetual, irrevocable, royalty-free basis to BMJ Publishing Group Ltd ("BMJ") its licensees and where the relevant Journal is co-owned by BMJ to the co-owners of the Journal, to publish the Work in this journal and any other BMJ products and to exploit all rights, as set out in our licence.

The Submitting Author accepts and understands that any supply made under these terms is made by BMJ to the Submitting Author unless you are acting as an employee on behalf of your employer or a postgraduate student of an affiliated institution which is paying any applicable article publishing charge ("APC") for Open Access articles. Where the Submitting Author wishes to make the Work available on an Open Access basis (and intends to pay the relevant APC), the terms of reuse of such Open Access shall be governed by a Creative Commons licence – details of these licences and which Creative Commons licence will apply to this Work are set out in our licence referred to above.

Other than as permitted in any relevant BMJ Author's Self Archiving Policies, I confirm this Work has not been accepted for publication elsewhere, is not being considered for publication elsewhere and does not duplicate material already published. I confirm all authors consent to publication of this Work and authorise the granting of this licence.

For peer review only

Why "one size fits all" is not enough when designing COVID-19 immunity certificates for domestic use: a UK wide cross-sectional online survey

Corina-Elena Niculaescu

Department of Computer Science, Brunel University London, UK, ORCID 0000-0002-8971-0812

Isabel Karen Sassoon

Department of Computer Science, Brunel University London, UK, ORCID 0000-0002-8685-1054

Irma Cecilia Landa-Avila

School of Design and Creative Arts, Loughborough University, UK, ORCID 0000-0001-6107-6736

Ozlem Colak

School of Design and Creative Arts, Loughborough University, UK, ORCID 0000-0003-0813-2561

Gyuchan Thomas Jun

School of Design and Creative Arts, Loughborough University, UK, ORCID 0000-0002-0958-0107

Panagiotis Balatsoukas

School of Design and Creative Arts, Loughborough University, UK, ORCID 0000-0002-2454-0816

Correspondence to Dr Isabel Sassoon Isabel.sassoon@brunel.ac.uk

For peer review only - http://bmjopen.bmj.com/site/about/guidelines.xhtml

Abstract

Objectives: The present study explored public's willingness to use COVID-19 immunity certificates across six different domestic scenarios.

Design: Cross-sectional online survey.

Setting: UK representative survey conducted on the 3rd of August 2021.

Participants: 534 UK residents over 18 years old.

Interventions: Participants replied to the same set of questions.

Primary and secondary outcome measures: The primary outcome measure was *willingness to use* immunity certificates across three different domestic settings (1. Visiting the GP for a non-urgent health issue, 2. Dining in a restaurant, and 3. Attending a performance in a theatre). For each setting two options, one prioritising *convenience* (option A) and the other *privacy* (option B), were offered. Our secondary outcome measures were computed indices from items adapted from the Health Belief Model; Attitudes towards sharing immunity status with service providers; Prior to COVID-19 lifestyle. In addition, we recorded data about respondents' socio-demographic characteristics.

Results: Respondents were more willing to use immunity certificates that prioritised *convenience*, rather than *privacy,* when visiting their GP (92%). However, privacy was more favorable (84%) in the other two settings (dining in a restaurant and going to a theatre) compared to *convenience* (39%). Personal beliefs about COVID-19 and immunity certificates were associated with variations in willingness to use these across all scenarios. No variations were observed across socio-demographics and lifestyle.

Conclusions: The findings of this survey suggest that there is not *one size fits all* solution for designing immunity certificates. Immunity certificates are complex socio-technical systems, any attempt to implement these for domestic use should be tailored to different settings and user needs. The design of certification services requires a more evidence-based approach and further research is needed to understand how different settings, design elements (like *convenience* or *privacy*) and personal beliefs about the pandemic should inform their design.

Keywords: COVID-19, service design, public health, vaccine certificates, immunity certificates, survey

Word count: 4,641

For peer review only

Strengths and limitations of this study

- The study reports knowledge about the interaction between individual characteristics, domestic settings, and types of immunity certificate design on willingness to use these certificates.
- UK nationally representative sample for age, gender, and ethnic background, but limited to people who have the means and capacity to use digital technologies (survey administered using Prolific.co).
- Since, as to the writing of this paper, COVID-19 certification has not been mandated in the UK, the scenarios used in the survey were hypothetical.
- We employed a Generalised Linear Mixed Effects Model (GLMM) to analyse whether there was a significant difference in the likelihood of using option A (*convenience*) and B (*privacy*) in each of the three settings

For peer review only

For peer review only - http://bmjopen.bmj.com/site/about/guidelines.xhtml

1. Introduction

Since the beginning of the COVID-19 pandemic immunity or vaccine certificates and their adoption by the public for domestic use has sparked a debate. The source for this debate can be attributed to several factors such as the uncertainty around the concept of immunity itself (e.g. what does it really mean to be immune against COVID-19 and how long does this last?)[1–3], the almost antagonist tension between the protection of public health and the respect for human rights or civil liberties (e.g. potential for creating inequality between those who are "immunoprivileged" and those who are "immunodeprived") as well as loss of autonomy[2,4–8], legal challenges in implementing COVID-19 certification across different industries[9,10], risk of fraud[11] and identity theft[5], and fear of digital exclusion[12–14]. Nonetheless, alongside the aforementioned concerns some potential benefits of immunity certificates have been reported such as preserving freedom of movement[15], re-opening the economy[16], reducing risk of infection and social benefits from increased social engagement[12,16].

The debate on the above issues has been reported both among scientists and the public. In a survey of 12,738 scientists from 63 countries, while roughly 22% of the respondents reported concerns around inequality, more than half of them agreed that immunity certificates would be beneficial for the economy and public health[16]. In a different study, textual analysis of Twitter sentiment also showed that in the UK and US vaccine certificates were associated with positive points among the public such as economic recovery, return to normality, safety, return to work or international travel, alongside negative connotations of discrimination[14], surveillance or civil liberties[17]. Similar were the findings reported by another UK nationwide questionnaire survey exploring public attitudes towards vaccine passports[18]. The findings of this survey also showed that people from ethnic minority backgrounds and lower income (less than £20K) would feel unfairly discriminated from the use of vaccine passports.

In the present study we approach immunity certification as a complex socio-technical system. Unlike previous published research we hypothesize that in order to understand what is the best way to design immunity certificates for domestic use we need to investigate the role of different contextual and situational factors, including different types of designs for COVID-19 certification, settings, individual characteristics and their interaction.

The present paper aims to progress the conversation around COVID-19 certification by answering two main research questions. **First, would a design that promotes *convenience* or *privacy* increase willingness to use immunity certificates across three different domestic settings (1. visiting the GP for a non-urgent health issue, 2. Dining in a restaurant, and 3. Attending a performance in a theatre)? Second, what is the role of socio-demographic characteristics, lifestyle, attitudes towards service providers and personal beliefs about COVID-19 on the**

For peer review only - http://bmjopen.bmj.com/site/about/guidelines.xhtml

aforementioned question? To address these two questions, we ran a nationally representative online questionnaire survey in the United Kingdom. Our findings produced unique knowledge about the interaction between individual characteristics, settings of use and types of certificate design on willingness to use immunity certificates. This knowledge can inform current policy on COVID-19 certification and lead to further development of existing solutions for domestic use, such as the UK's NHS COVID Pass[19].

In the present paper the term "immunity certificate" refers to a type of certification (digital or paper) showing that an individual has developed antibodies of SARS-COV-2 either through past infection or after completing a full course of vaccination. We chose to focus on the concepts of *convenience* and *privacy* because both concepts have been reported frequently in the literature as key factors of user experience influencing use and/or adoption of these certificates among the public[13,20–24]. Purposefully, we selected to present *convenience* and *privacy* as two extreme options to understand how willingness to use immunity certificates may be affected across different settings and individual characteristics. This decision was influenced by the findings of our previous studies where research participants perceived the two concepts not as complementary components to the design of services for immunity certificates but as antagonist elements that bring tension and dilemmas[25]. Therefore, in the context of the present study, the ***privacy*** option involved an individual installing the NHS app, accessing the COVID-19 certificate, generating or downloading a 2D barcode and presenting this to the service provider for validation. In this option the user of the service does not share any personal data electronically and the service provider, for example the restaurant, only scans the 2D barcode and manually checks the details in the certificate against the individual's form of identification. On the other hand, we hypothesised ***convenience*** as a situation where the individual would not need to download or install an app and generate or download and share 2D barcodes. In the case of this scenario we hypothesised that it would be more convenient for individuals to share their NHS number with the service provider. Then the service provider would use this number to verify someone's immunity status directly with the NHS (for instance, by checking it against the records held in the National Immunisation Management System NIMS). For example, when visiting a theatre to watch a performance the theatre company will verify a customer's immunity status directly with the NHS using the customer's NHS number. In both cases it is the responsibility of the service provider to validate an individual's immunity status but in the case of the *privacy* option the customer or service user needs to go through a process that requires more physical and cognitive effort, while in the case of the convenience option the individual only shares their NHS number (without the need to install any apps or generate and share barcodes). In the case of the convenience option the individual is required to share personal information (i.e. NHS number) with the service provider, while in the

For peer review only - http://bmjopen.bmj.com/site/about/guidelines.xhtml

case of the privacy option the service provider only validates the generated or downloaded barcode without digitally processing personal information (like the NHS number)[26].

Finally, we selected to focus on the specific three domestic settings (GP appointment for a non-urgent health matter, dining in a restaurant and going to a theatre) because all three represent common, yet distinct (in terms of purpose and social behaviours evoked), types of services that take place indoors.

2. Methods

2.1. Design & Methods

Our analysis was based on a cross-sectional dataset generated from an online questionnaire survey that took place on the 3rd of August 2021. The online questionnaire was created using the *onlinesurveys.ac.uk* platform and administered via *prolific.co.* All the materials related to this survey including survey instrument, raw dataset, statistical code, and ethics approval are available on OSF (https://osf.io/jubv6/ DOI:10.17605/OSF.IO/JUBV6).

2.1.1. Sample Design

Respondents were demographically representative of the UK population in terms of gender, age, and ethnicity. Summary statistics for all demographic variables can be found in the Supplementary Material. We excluded 20 participants who failed the attention checks, and one duplicate responder, resulting in a final sample of 534 respondents. All participants were 18 or older and were compensated for their participation in the study with £1.75/person.

2.1.2. Patient and Public Involvement

The research questions and contents of the survey were informed by the findings of a series of studies (composed of focus groups and interviews) which involved a diverse group of research participants including patients, members of the public and service providers from the cultural, sports, hospitality and aviation sectors[25].

2.2. Main variables measure

2.2.1. Willingness to use Immunity Certificates across different scenarios (Primary Outcome)

Each scenario was presented in the form of a short narrative description of a hypothetical use case that combined one of the three settings of interest, as described in section 1, (1. Visiting the GP for a non-urgent health issue; 2. Dining in a restaurant; 3. Attending a performance in a theatre), with one of the following two options: A. Convenience; And B. Privacy. Since the process of using COVID-19 certification for domestic purposes has not been mandated to date in the UK, and there is lots of speculation about how these could become operational in practice, the six

For peer review only - http://bmjopen.bmj.com/site/about/guidelines.xhtml

scenarios explored hypothetical or future situations balancing imagination/creativity with rigorous reasoning techniques[25,27]. For illustration purposes, Figure 1 presents the six scenarios. In the case of the *convenience* option the service providers in each setting (e.g. restaurant management or theatre company) had the authority to check a customer's COVID-19 immunity status with the NHS, without any additional steps on behalf of the users to prove their status. In the other option (*privacy option*) according to the scenario users needed to generate and share a QR code upon booking an appointment or making a reservation with the service provider to demonstrate their immunity status. In this case, users were actively in control of the process of sharing their immunity status but they had to perform a series of actions to generate/obtain and share the QR code[20,25].

[Figure 1]

In each of the six scenarios willingness to use immunity certificates was measured by asking respondents to rate how likely they were to use these based on a 5-point Likert Scale (ranging from "Extremely Unlikely" to "Extremely Likely"). Following the data collection, positive to neutral answers, including "Extremely Likely" "Somewhat Likely" and "Neither Likely nor Unlikely" were grouped together under "Yes" describing willingness to use the service. Negative answers including "Extremely Unlikely" and "Somewhat Unlikely" were grouped together under "No" describing reluctance to use the service. We dichotomised the scale this way to make its interpretation easier, and to differentiate between those willing to use the service (neutral to positive answers) and those resistant (negative answers). A similar transformation of 5-Point Likert scale likelihood variables was used in previously published research reporting results from a series of surveys on adherence to test, trace and isolation measures in the UK[28].

2.2.2. Health-Belief Model (Secondary Outcome)

We used a selection of items adapted from the Health Belief Model (HBM)[29] to examine whether certain health beliefs towards vaccination and COVID-19 could influence respondents' willingness to use immunity certificates across the six scenarios. The detailed description of the items, summary statistics and internal reliability measures are presented in Table 1. Each item was rated on a 5-point Likert scale from 1 ("Strongly disagree") to 5 ("Strongly agree"). First, we measured *COVID-19 Susceptibility* (respondent's perceived susceptibility) using three items adapted from[30] and one item from[31]. Second, we measured *Certificate Severity* (as the perceived severity of not using immunity certificates) using an index of six items. Additional HBM constructs were measured in our survey, but their analysis is beyond the scope of this paper and therefore these are not shown in Table 1.

For peer review only - http://bmjopen.bmj.com/site/about/guidelines.xhtml

Table 1 Summary statistics and reliability of HBM measures, and willingness to share immunity status

	Survey Items	Mean	Median	Std. Dev	Min	Max	Alpha
COVID-19 Perceived Susceptibility (HBM)	I am at risk of getting COVID-19 (SARS-CoV-2).	3.5243	4	1.1255	1	5	0.7095
	It is likely that I will get COVID-19 (SARS-COV-2).	2.9401	3	1.0122	1	5	
	Individuals in my household are at risk for getting COVID-19 (SARS-COV-2).	3.4438	4	1.1310	1	5	
	I feel knowledgeable about my risk of getting COVID-19 (SARS-COV-2).	4.1255	4	0.7460	1	5	
Certificate Severity (HBM)	I feel that without this service I won't be able to return to my workplace.	2.4476	2	1.1558	1	5	0.8485
	I feel that without this service my chances of getting a job will be affected.	2.5918	3	1.1631	1	5	
	I feel that without this service I won't be able to book face-to-face appointments with my GP/dentist.	2.8371	3	1.2455	1	5	
	I feel that without this service I won't be able to go to the theatre/movies/sports events.	3.2715	4	1.1636	1	5	
	I feel that without this service I won't be able to travel internationally.	3.912	4	1.1252	1	5	
	I feel that without this service I will not enjoy the same liberties I did before the pandemic.	3.6667	4	1.1692	1	5	
Willingness to Share Immunity Status with Service Providers	Theatre/cinema/gallery	3.2921	4	1.3998	1	5	-
	Pub/restaurant.	3.2228	4	1.4159	1	5	
	GP/dentist	4.47	5	0.9219	1	5	

For peer review only - http://bmjopen.bmj.com/site/about/guidelines.xhtml

	Hospitality sector	3.4663	4	1.3717	1	5	
	Sports event	3.3015	4	1.4012	1	5	
	Airport/airline	3.8764	4	1.2538	1	5	

As it is shown in Table 1, *COVID-19 Susceptibility* generated a Cronbach's Alpha of 0.7095 and Certificate Severity a score of 0.8485 suggesting good internal consistency[32]. Therefore, we created an index (*Certificate Severity;* and *COVID-19 Susceptibility)* for each of these two constructs by averaging the items within the constructs[20]. Then we used these indices to explore whether there were potential factors influencing willingness to use immunity certificates across the different scenarios.

2.2.3. General attitudes towards sharing Immunity Status with Service Providers (Secondary Outcome)

Respondents were asked also to rate their willingness to share their immunity status with the following types of service providers: primary care GP/dentist, airport/airline, hospitality (pub/restaurant and hotel), cultural and creative industries (theatres/cinemas/galleries) and sports event organisers (such as football clubs). Responses were recorded using a 5-point Likert scale ranging from 1 ("Strongly disagree") to 5 ("Strongly agree"). We used these items to examine whether willingness to use immunity certificates measured by our primary outcome was affected by willingness to share immunity status with different service providers in general.

2.2.4. Prior to COVID-19 Lifestyle (Secondary Outcome)

We asked a series of lifestyle questions to determine if respondents' habits before the COVID-19 outbreak correlated with the willingness to use immunity certificates in the different scenarios. Lifestyle questions recorded the respondents' perceived frequency of attending or pursuing various activities of immediate interest to the scenarios under investigation including going to the theatre or other cultural venues (like museums and galleries), going to pubs, restaurants, and other dining venues, or visiting health care settings. Other lifestyle questions collected data about their frequency of travelling abroad, and booking accommodation when travelling abroad, Responses were captured using a 4-point Likert scale ranging from 1 ("Never") to 4 ("Very often").

2.3. Statistical Analysis

To address the first research question, this survey design enabled us to analyse the responses collected for the primary outcome measure (i.e., willingness to use immunity certificates across different scenarios) as a repeated measures 2*3 factorial design with two fixed effects (the *convenience/privacy options* and the setting) and with a random effect of the responder. The dependent variable for this analysis was the willingness to use immunity certificates. Each responder had six willingness to use responses each corresponding to a different scenario which was the result of the combination of *convenience* versus *privacy* options and settings[33].

For peer review only - http://bmjopen.bmj.com/site/about/guidelines.xhtml

To analyse whether there was a significant difference in the likelihood of using option A (*convenience*) and B (*privacy*) in each of the three settings we employed a Generalised Linear Mixed Effects Model (GLMM)[33,34]. We fit the GLMM model which incorporates both fixed effects parameters (*convenience/privacy* and setting) and random effects in a linear predictor, via maximum likelihood.

Finally, to address our second research question, we employed an exploratory analysis through graphical representations of the relationships between the dependent variables (primary outcome measure) and the other secondary outcome measures. To display relationships between the primary outcome measure and the HBM indices we used box plot graphs, and for the rest of the variables we present mosaic plots. The statistical analysis was performed in STATA[35] and R[36].

2.4. Power calculation

The sample size was chosen pragmatically based on several different approaches, obtaining a minimum sample size between 271 and 1,067 participants, depending on the assumptions. This sample size results in a 99% power in the GLMM model used in our statistical analysis of the first research question.

3. Results

3.1. Does a person's willingness to use immunity certificates vary across the six scenarios?

Figure 2 illustrates the distribution of responses across the six scenarios while Table 2 the proportion of respondents who would be willing to use the certificates across these scenarios. The data show that the majority of respondents (92%) were more willing to use immunity certificates that prioritised *convenience* when visiting their GP (Scenario 1A). However, *convenience* was less favorable in the other two settings with only 38% and 39% of respondents willing to use the certificates for dinning indoors (Scenario 2A) and going to the theatre (Scenario 3A) respectively.

[Figure 2]

Table 2 Acceptance of different service designs and settings

Response	NO	YES
Scenario 1A: Visiting the GP/Convenience option	44	490 (92%)
Scenario 1B: Visiting the GP /Privacy option	129	405 (76%)
Scenario 2A: Dining in a Restaurant/Convenience option	329	205 (38%)
Scenario 2B: Dining in a Restaurant/Privacy option	85	449 (84%)
Scenario 3A: Attending a Performance in the Theatre/Convenience option	324	210 (39%)

For peer review only - http://bmjopen.bmj.com/site/about/guidelines.xhtml

Scenario 3B: Attending a Performance in the Theatre/Privacy option	89	445 (83%)

Table 3 Summary of the coefficients of the Generalized linear mixed model fit

Fixed Effects	Estimate	Std. Error	z value	Pr (>|z|)
(Intercept)	4.1636	0.2646	15.7300	<2e-16 ***
Dining in a Restaurant Setting	-5.0121	0.2837	-17.6700	<2e-16 ***
Attending a Performance in the Theatre Setting	-4.9398	0.2822	-17.5000	<2e-16 ***
Privacy Option	-2.0736	0.2504	-8.2800	<2e-16 ***
Dining in a Restaurant Setting Setting*Privacy Option	5.8747	0.3595	16.3400	<2e-16 ***
Attending a Performance in the Theatre Setting*Privacy Option	5.7127	0.3557	16.0600	<2e-16 ***

Note: Table 3 contains the coefficients in log-odds form of the GLMM model and their significance. The estimates of the fixed effects are conditional on the random effects. The estimated effects have a binary outcome with a logit link; hence the raw estimates are on the log-odds scale. The intercept refers to the log-odds for willingness to use immunity certificates in Scenario 1A (visiting the GP/Convenience option). A positive value for log-odds estimates respondents being likely to be willing to use the certificate in that setting and option. The coefficients on the log-odds scale are additive.
*Significance codes 0 '***'; 0.001 '**'; 0.01;'*'; 0.05 '.'; 0.1 ' '*

To determine whether these differences in willingness to use were statistically significant we applied the GLMM model, including fixed and random effects outlined in Section 2.3. Table 3 includes a subset of the R output for the GLMM analysis (logistic regression with mixed fixed and random effects). The estimated coefficient for the intercept 4.1636, is the log odds for scenario 1A (choosing *convenience* option for visiting the GP for a non-urgent health issue). The estimate of -5.0121 for dinning in a restaurant means that this setting is associated with a 5.0121 decrease in the log-odds of positive response ("Yes") compared to negative response ("No"). The log-odds for scenario 2B (choosing *privacy* for dining in a restaurant) is the sum of the intercept (4.1636), the indoor dining setting (-5.0121), the *privacy* option (-2.0736), and their interaction (5.8747), resulting in a log-odds of 2.9526. This points to a higher likelihood of willingness to use immunity certificates for scenario 2B (choosing *privacy* for dining in a restaurant) than for scenario 2A (choosing *convenience* for dining in a restaurant).

The likelihood of using the certificate when going a restaurant or to the theatre was significantly lower than visiting the GP with a statistically significant p-value<0.001 when considering the scenarios irrespective of the option. Also, the likelihood of using the certificate was significantly lower for *privacy* than *convenience* when visiting the GP (p-value<0.001). However, when

For peer review only

For peer review only - http://bmjopen.bmj.com/site/about/guidelines.xhtml

considering the *privacy option* in the restaurant or theatre setting, this likelihood of using the certificate is higher and statistically significant (p-value<0.001).

3.2. What is the role of personal beliefs about COVID-19 (certificate severity and COVID-19 susceptibility), attitudes towards service providers, lifestyle, and socio-demographic characteristics on the willingness to use immunity certificates?

To address our second research question, we graphically explored the primary outcome variables against all other secondary outcome variables, but for brevity we only present here the key results.

- *Certificate severity*. Lower values for *Certificate Severity* suggest that respondents did not perceive immunity certificates as necessary (Figure 3A). We observed lower perceived *Certificate severity* among those who were not willing to use immunity certificates. This finding was observed across all settings and for both options (*convenience* and *privacy*).

[Figure 3]

- *COVID-19 susceptibility*. The median of *perceived susceptibility* is consistently higher for responses indicating willingness to use immunity certificates across all scenarios even when these prioritised *convenience* (Figure 3B). This means that respondents who perceived themselves as being more susceptible to COVID-19 were more willing to use immunity certificates for both options. Also, the median values show that this group of respondents would be more willing to use an option that prioritised *convenience* when going to a restaurant or the theatre rather than when visiting their GP for a non-urgent health matter. This finding suggests that people who perceived themselves to be at high risk from COVID-19 were more willing to trade-off *privacy* for *convenience* for specific settings.

- *General attitudes towards sharing immunity status with service providers*. Respondents who were comfortable sharing their COVID-19 immunity status with service providers from the cultural and creative industries (e.g. theatres, cinemas or galleries) were more willing to use immunity certificates across all three settings (Figure 4). Also, this group of respondents was more likely to choose options prioritising *convenience*. On the contrary respondents who did not like sharing their immunity status with service providers from the creative and cultural industries were less willing to use immunity certificates across all scenarios even when this prioritised *privacy*. Similar patterns with those exhibited in the case of cultural and creative industries were observed in the case

For peer review only - http://bmjopen.bmj.com/site/about/guidelines.xhtml

of the other types of service providers, including hospitality, airlines/airports, sports events, and pubs/restaurants.

[Figure 4]

- *The role of socio-demographics and lifestyle characteristics.* Our exploration of the relationship between willingness to use the immunity certificates across the six scenarios did not point to any significant differences across socio-demographics or lifestyle. Specifically, we observed no variation in willingness to use immunity certificates across the different scenarios by gender, age, and ethnicity. Also, willingness to use immunity certificates did not vary by other socio-demographics such as disability, living arrangements and living in a rural versus urban area. In addition, lifestyle characteristics were not associated with variations in willingness to use immunity certificates either. Lastly, there was no variation in willingness to use the service based on mental wellbeing and net income now as compared to before the outbreak, suggesting that willingness to use immunity certificates does not stream from feelings of hopelessness.

4. Discussion

Practical implication for policy making

As of the writing of this paper (September 2021), the use of immunity certificates for domestic purposes in the UK was not mandatory. Also, there was still lack of knowledge or guidance about what is the best way to design immunity certificates for use in different domestic settings. Therefore, the present survey contributes unique knowledge that different stakeholders (the NHS, the UK government and the Scientific Advisory Group for Emergencies, businesses, and the public) should take into account when considering the use of immunity certificates for domestic purposes. Some key implications of this study for policy making are the following:

- *Use of certification in primary care settings*. Participants' responses showed high willingness to use immunity certificates when visiting their GP for a non-urgent health matter compared to the other settings (dinning in a restaurant and going to the theatre). In this case the majority of responses were positive for both options (*privacy* and *convenience*). We argue that the implementation of immunity certificates for non-urgent healthcare matters could play a role in increasing the sense of safety as well as reducing waiting times for face-to-face appointments especially when the option that prioritises *convenience* is selected to confirm patients' immunity status.

  However, as of today, in the UK, the use of any type of immunity certificate in healthcare settings is not recommended. Currently consultations with a GP can be either remote (videoconferencing or via telephone) or face-to-face. The transition to pre-pandemic

For peer review only - http://bmjopen.bmj.com/site/about/guidelines.xhtml

appointments is not fully completed, and the risks of attending face-to-face appointments are still present. For instance, dental procedures can increase the risk of SARS-CoV-2 transmission due to tools that produce aerosols[37,38], not to mention the issue of contracting the virus in a hospital setting[39,40]. As such patients and health care professionals are presented with the choice between remote appointments for non-urgent matters or the risk of infection in a face-to-face setting. While to a certain extend there is some evidence that remote consultations have been accepted well by the patients[41], several challenges have been reported too, especially in the case of people who suffer from pre-existing chronic conditions or who they may not feel comfortable with the use of technology[41–43]. For those patients, implementation of immunity certificates for face-to-face appointments would be important in improving their sense of safety. In addition, our findings showed that as opposed to the other domestic settings, in the case of visiting a GP for a non-urgent matter, respondents were more willing to use immunity certificates that prioritised *convenience.* The reason for choosing this option could be justified by the fact that respondents valued the public health benefits of using certificates in this context as more important compared to the option than prioritised privacy[44]. Also, this means that respondents in this survey preferred access to primary care services to be seamless without a need for patients to show proof of their immunity status in a digital or physical format. Instead, this proof could be verified by the GP practice upon booking an appointment (over the phone or electronically). This would result in zero checks at the reception, shorter queues upon arrival and better experience for the patients.

- *Use of certification for social indoor activities.* When it comes to leisure activities (such as dining in a restaurant or going to a theatre) respondents were less willing to use immunity certificates, and if they did most of them would value the *privacy* option over *convenience*. One key implication of this finding when designing certification services is to make clear to the public how data *privacy* and protection applies to this context. The findings of our previous research[25] suggest that the public is not convinced about whether or not their data is shared when using these certificates, thus making them more sceptical to use these in settings where they feel that they are less secure or they do not trust. This happens despite the fact that in the UK verification of someone's immunity status using the paper or digital certification format (via the NHS app[19]) does not involve sharing of any personal information with the service provider (for example, the theatre company or the restaurant management team can only verify the validity of the barcode itself)[45]. For the use and uptake of such certificates to be successful the public and businesses should be educated, and nation-wide public health campaigns should promote this shared understanding explaining the extent to which personal data, if any, is

For peer review only - http://bmjopen.bmj.com/site/about/guidelines.xhtml

shared upon verification of someone's immunity status. Researchers examining the implementation of the European Union's COVID digital certificate for international travel (known as green pass) highlighted the importance of campaigns to educate the public about the purpose, opportunities and limitations of these certificates too[44]. In addition to educating the public and promoting the importance of certification for public health, the presence of such nation-wide campaigns is important for transparency reasons too. Different countries around the globe have used immunity certificates in ways that made possible the collection and processing of large amounts of personal data about individuals for reasons other than merely proving their immunity status, for example, in order to monitor the flow of people in shops or other settings[46]. Explaining and communicating decisions and actions around public health to the public in a transparent way has been identified as a key leadership characteristic of public health authorities that was lacking during the first wave of this pandemic both in the EU[47] and the UK[48].

- *Need to build empathy and understand public's views about COVID-19 when designing certification services*. While lots of attention when designing different forms of COVID-19 certification was placed on issues surrounding their accessibility[14,49–51], less effort has been put in place to harness public's beliefs around COVID-19 and COVID-19 certification and fed these into the design of services for immunity certificates. The implementation of immunity certificates should be accompanied by a series of health promotion strategies tailored to target the needs of people with different beliefs, knowledge and understanding about COVID-19, and ultimately change their behaviour. The present survey showed that traditional demographic information and lifestyle does not influence user willingness to use immunity certificates across the six scenarios. However, our findings also showed that perceived risk of falling severely ill from COVID-19 (*COVID-19 susceptibility*) and perceived severity from not using immunity certificates (*Certificate severity*) can influence public's willingness to use immunity certificates.

Limitations

One of the limitations of our study is that participants were recruited using the online survey platform Prolific. Since surveys administered via this platform are completed online (mobile, PC, tablet etc.) our sample is comprised of people who had the means and capacity to use digital technologies. Other studies investigating people's perceptions of immunity certificates[14,20,52] or COVID-19 vaccine intentions[28,53–55] found some differences based on gender and ethnicity, which we did not find. This can be explained by the fact that unlike the other cited studies, our survey was focused on six specific hypothetical scenarios of using immunity certificates. Another possible explanation can be attributed to the timing of this survey. At the time our survey took

For peer review only - http://bmjopen.bmj.com/site/about/guidelines.xhtml

place immunity certificates were being used for international travel, hence access, awareness and familiarity with such services were higher than in previously published studies. Finally, from a methodological point of view the mixed effects model we used assumed that the random effect subsumes the possible effects of gender and ethnicity, as we are looking at responses of the same person. Therefore, both the scope and timing of the present study as well as the mixed effects model used differed from other surveys in this context.

5. Conclusions

The findings of this survey suggest that there is not *one size fits all* solution for designing immunity certificates. Immunity certificates should be studied as complex socio-technical services rather than merely products that one can simply download and use. Any attempt to implement such certificates for domestic use should be tailored to different settings and user needs. While some implications of our findings for policy making were discussed, the design of certification services requires a more evidence-based approach and further research is needed to examine willingness to use immunity certificates across the present three, and possibly other, domestic settings. Also, while in the present survey we chose to explore two options for designing immunity certificates (one prioritising *convenience* and the other *privacy*), our previous work[56] has shown that there are still more attributes that may influence use in this context and future research should focus on.

For peer review only - http://bmjopen.bmj.com/site/about/guidelines.xhtml

Footnotes

Contributors: The questionnaire survey was conceptualised by CN, IS and PB, with the input of TG, CLA, and OC. CEN and PB completed the data collection. CN and IS conducted the statistical analysis. All authors contributed and approved the final manuscript.

Funding: IMMUNE or Immunity Passport Service Design is a nine-month project funded by the AHRC/UKRI COVID-19 Rapid Response (Ref. AH/W000288/1).

Ethics statements: Ethics approval was obtained from the College of Engineering, Design and Physical Sciences Research Ethics Committee at Brunel University London (Ref. 31705-A-Jul/2021- 33586-1) on the 29th of July 2021.Informed consent was obtained from all respondents prior to the beginning of the survey. Respondents were allowed to withdraw from the survey at any time.

Data availability statement: The data is available in a public, open access repository. All statistical code, dataset, survey item and ethical approval are available on OSF (https://osf.io/jubv6/), DOI 10.17605/OSF.IO/JUBV6.

Competing interests: None declared.

For peer review only

References

1 Wilf-Miron R, Myers V, Saban M. Incentivizing Vaccination Uptake: The 'green Pass' Proposal in Israel. *JAMA - J Am Med Assoc* 2021;**325**:1503–4. doi:10.1001/jama.2021.4300

2 Larremore DB, Bubar KM, Grad YH. Implications of Test Characteristics and Population Seroprevalence on 'Immune Passport' Strategies. *Clin Infect Dis* 2021;**72**:e412–4. doi:10.1093/cid/ciaa1019

3 Liew CH, Flaherty GT. Immunity passports to travel during the COVID-19 pandemic: controversies and public health risks. *J Public Health (Oxf)* 2021;**43**:e135–6. doi:10.1093/pubmed/fdaa125

4 Voo TC, Reis AA, Thomé B, *et al.* Immunity certification for COVID-19: Ethical considerations. *Bull World Health Organ* 2021;**99**:155–61. doi:10.2471/BLT.20.280701

5 Liz J. COVID-19, immunoprivilege and structural inequalities. *Hist Philos Life Sci* 2021;**43**:1–6. doi:10.1007/s40656-020-00356-5

6 Bramstedt KA. Antibodies as Currency: COVID-19's Golden Passport. *J Bioeth Inq* Published Online First: 2020. doi:10.1007/s11673-020-09996-5

7 Brown RCH, Savulescu J, Williams B, *et al.* Passport to freedom? Immunity passports for COVID-19. *J Med Ethics* 2020;**46**:652–9. doi:10.1136/medethics-2020-106365

8 Kosciejew MRH. COVID-19 immunity (or vaccine) passports: a documentary overview and analysis of regimes of health verification within the coronavirus pandemic. *J Doc* 2021;**ahead-of-p**. doi:10.1108/JD-04-2021-0079

9 Voo TC, Clapham H, Tam CC. Ethical implementation of immunity passports during the COVID-19 pandemic. *J Infect Dis* 2020;**222**:715–8. doi:10.1093/infdis/jiaa352

10 Phelan AL. COVID-19 immunity passports and vaccination certificates: scientific, equitable, and legal challenges. *Lancet* 2020;**395**:1595–8. doi:10.1016/S0140-6736(20)31034-5

11 Rocha ICN. How to avoid fake COVID-19 vaccine passports as a travel requirement? *J Public Health (Oxf)* 2021;:1–2. doi:10.1093/pubmed/fdab308

12 HÄKLI J. COVID-19 Certificates as a New Form of Mobility Control. *Eur J Risk Regul* 2021;:1–8. doi:10.1017/err.2021.21

13 WILFORD SH, MCBRIDE N, BROOKS L, *et al.* The Digital Network of Networks:

For peer review only - http://bmjopen.bmj.com/site/about/guidelines.xhtml

Regulatory Risk and Policy Challenges of Vaccine Passports. *Eur J Risk Regul* 2021;:1–11. doi:10.1017/ERR.2021.35

14 Ada Lovelace Institute. The data divide: Public attitudes to tackling social and health inequalitites in the COVID-19 pandemic and beyond. 2021.

15 Persad G, Emanuel EJ. The Ethics of COVID-19 Immunity-Based Licenses ("Immunity Passports"). *JAMA* 2020;**323**:2241–2. doi:10.1001/jama.2020.8102

16 Aranzales I, Chan HF, Eichenberger R, *et al.* Scientists have favorable opinions on immunity certificates but raise concerns regarding fairness and inequality. *Sci Rep* 2021;**11**:14016. doi:10.1038/s41598-021-93148-1

17 Uk W, Think R, Passport AV. Have Vaccine Passport Will Travel: What UK Residents Think About Vaccine Passport. 2021.

18 Ada Lovelace Institute. Public attitudes towards vaccine passports. 2021.https://www.adalovelaceinstitute.org/survey/public-attitudes-towards-vaccine-passports/ (accessed 10 Sep 2021).

19 Department of Health and Social Care. NHS COVID Pass. 2021.https://www.gov.uk/guidance/nhs-covid-pass (accessed 27 Sep 2021).

20 Lewandowsky S, Dennis S, Perfors A, *et al.* Public acceptance of privacy-encroaching policies to address the COVID-19 pandemic in the United Kingdom. *PLoS One* 2021;**16**:1–23. doi:10.1371/journal.pone.0245740

21 Hasan HR, Salah K, Jayaraman R, *et al.* Blockchain-Based Solution for COVID-19 Digital Medical Passports and Immunity Certificates. *IEEE Access* 2020;**8**:222093–108. doi:10.1109/ACCESS.2020.3043350

22 Ricci L, Di Francesco Maesa D, Favenza A, *et al.* Blockchains for covid-19 contact tracing and vaccine support: A systematic review. *IEEE Access* 2021;**9**:37936–50. doi:10.1109/ACCESS.2021.3063152

23 GSTREIN OJ. The EU Digital COVID Certificate: A Preliminary Data Protection Impact Assessment. *Eur J Risk Regul* 2021;:1–12. doi:10.1017/err.2021.29

24 Papadaki M, Karamitsos I, Themistocleous M. ViewpointCovid-19 digital test certificates and blockchain. *J Enterp Inf Manag* 2021;**34**:993–1003. doi:10.1108/JEIM-07-2021-554

25 Landa-Avila IC, Thomas Gyuchan J, Sassoon I, *et al.* COVID-19 Immunity certificates as complex systems: Applying systems approaches to explore needs, risks, and unintended consequences. In: *Relating Systems Thinking & Design Symposium RSD10*. RSD10 2021.

For peer review only - http://bmjopen.bmj.com/site/about/guidelines.xhtml

26 Wolford B. A guide to GDPR data privacy requirements. GDPR EU. https://gdpr.eu/data-privacy/

27 Ramirez R, Mukherjee M, Vezzoli S, *et al.* Scenarios as a scholarly methodology to produce 'interesting research'. *Futures* 2015;**71**:70–87. doi:10.1016/j.futures.2015.06.006

28 Smith LE, Potts HWW, Amlôt R, *et al.* Adherence to the test, trace, and isolate system in the UK: results from 37 nationally representative surveys. *bmj* 2021;**372**.

29 Champion VL, Skinner CS. The health belief model. In: *Health behavior and health education: Theory, research, and practice, 4th ed.* San Francisco, CA, US: : Jossey-Bass 2008. 45–65.

30 Coe AB, Elliott MH, Gatewood SBS, *et al.* Perceptions and predictors of intention to receive the COVID-19 vaccine. *Res Soc Adm Pharm* 2021;:1–7. doi:10.1016/j.sapharm.2021.04.023

31 Chu H, Liu S. Integrating health behavior theories to predict American's intention to receive a COVID-19 vaccine. *Patient Educ Couns* Published Online First: 2021. doi:10.1016/j.pec.2021.02.031

32 Cortina JM. What is coefficient alpha? An examination of theory and applications. *J Appl Psychol* 1993;**78**:98–104. doi:10.1037/0021-9010.78.1.98

33 Agresti A. *Categorical data analysis*. Vol. 482. John Wiley & Sons. 2003.

34 Bates D, Maechler M, Bolker B, *et al.* Mixed-Effects Models Using lme4. *J Stat Softw* 2015;**67**:1–48. doi:10.18637/jss.v067.i01

35 StataCorp. Stata Statistical Software: Release 17. 2021.https://www.stata.com/

36 R Core Team. R: A language and environment for statistical computing. 2021.https://www.r-project.org/

37 Epstein JB, Chow K, Mathias R. Dental procedure aerosols and COVID-19. *Lancet Infect Dis* 2021;**21**:e73. doi:10.1016/S1473-3099(20)30636-8

38 Ge Z yu, Yang L ming, Xia J jia, *et al.* Possible aerosol transmission of COVID-19 and special precautions in dentistry. *J Zhejiang Univ Sci B* 2020;**21**:361–8. doi:10.1631/jzus.B2010010

39 Duncan C. Up to 8,700 patients died after catching Covid in English hospitals, figures show. Indep. 2021.https://www.independent.co.uk/news/health/covid-deaths-in-hospitals-b1853184.html

For peer review only

For peer review only - http://bmjopen.bmj.com/site/about/guidelines.xhtml

40 Oliver D. David Oliver: Deaths from hospital acquired covid are everyone's problem. *BMJ* 2021;**373**:372–3. doi:10.1136/bmj.n1492

41 Nijhof D, Ingram A, Ochieng R, *et al.* Examining GP online consultation in a primary care setting in east midlands , UK. 2021;:1–10.

42 Quinn-Scoggins HD, Cannings-John R, Moriarty Y, *et al.* Cancer symptom experience and help-seeking behaviour during the COVID-19 pandemic in the UK: A cross-sectional population survey. *BMJ Open* 2021;**11**:1–15. doi:10.1136/bmjopen-2021-053095

43 Hearn JH, Rohn EJ, Monden K. Isolated and anxious: A qualitative exploration of the impact of the COVID-19 pandemic on individuals living with spinal cord injury in the UK. *J Spinal Cord Med* 2021;**0**:1–9. doi:10.1080/10790268.2021.1949562

44 Montanari Vergallo G, Zaami S, Negro F, *et al.* Does the EU COVID Digital Certificate Strike a Reasonable Balance between Mobility Needs and Public Health? *Medicina (Kaunas)* 2021;**57**:1077. doi:10.3390/medicina57101077

45 Balatsoukas P, Jun GT, Sassoon I. Vaccine passports: what businesses need to know – and why they should have more say. 2021.https://theconversation.com/vaccine-passports-what-businesses-need-to-know-and-why-they-should-have-more-say-164606?utm_source=twitter&utm_medium=bylinetwitterbutton

46 Wang Q, Su M, Zhang M, *et al.* Integrating Digital Technologies and Public Health to Fight Covid-19 Pandemic: Key Technologies, Applications, Challenges and Outlook of Digital Healthcare. *Int J Environ Res Public Health* 2021;**18**. doi:10.3390/ijerph18116053

47 Gontariuk M, Krafft T, Rehbock C, *et al.* The European Union and Public Health Emergencies: Expert Opinions on the Management of the First Wave of the COVID-19 Pandemic and Suggestions for Future Emergencies . Front. Public Heal. . 2021;**9**.https://www.frontiersin.org/article/10.3389/fpubh.2021.698995

48 Adebowale V, Alderson D, Burn W, *et al.* Covid-19: Call for a rapid forward looking review of the UK{\textquoteright}s preparedness for a second wave{\textemdash}an open letter to the leaders of all UK political parties. *BMJ* 2020;**369**. doi:10.1136/bmj.m2514

49 GANTY S. The Veil of the COVID-19 Vaccination Certificates: Ignorance of Poverty, Injustice towards the Poor. *Eur J Risk Regul* 2021;:1–12. doi:10.1017/err.2021.23

50 Osama T, Razai MS, Majeed A. Covid-19 vaccine passports: access, equity, and ethics. *BMJ* 2021;**373**:n861. doi:10.1136/bmj.n861

51 The Royal Society. Twelve criteria for the development and use of COVID-19 vaccine

For peer review only - http://bmjopen.bmj.com/site/about/guidelines.xhtml

passports. 2021.

52 Hall MA, Studdert DM, Favorito L, *et al.* U.S. Public Views about COVID-19 ‘Immunity Passports’. *medRxiv* Published Online First: 2021. doi:10.1101/2021.01.26.21250184

53 Guidry JPD, Laestadius LI, Vraga EK, *et al.* Willingness to get the COVID-19 vaccine with and without emergency use authorization. *Am J Infect Control* 2021;**49**:137–42. doi:10.1016/j.ajic.2020.11.018

54 Paul E, Steptoe A, Fancourt D. Attitudes towards vaccines and intention to vaccinate against COVID-19: Implications for public health communications. *Lancet Reg Heal - Eur* 2021;**1**:100012. doi:10.1016/j.lanepe.2020.100012

55 Blundell R, Costa Dias M, Joyce R, *et al.* COVID-19 and Inequalities*. *Fisc Stud* 2020;**41**:291–319. doi:10.1111/1475-5890.12232

56 Landa-Avila IC, Colak O, Niculaescu CE, *et al.* Mapping COVID-19 Health Certificates as a Complex System (version 1.1). 2021. doi:10.17028/rd.lboro.14933391.v1

For peer review only

Figure Legends

Figure 1 Description of the six scenarios (the number represents one of the three settings while the letter the design option, convenience or privacy)

Figure 2 Distribution of number of responses (N) across settings (visiting the GP, dining in a restaurant, attending a performance in the theatre) and design options (convenience/privacy).

Figure 3. **A.** Index of Certificate Severity (perceived severity of not using immunity certificates) and **B**. Index of COVID-19 Susceptibility across settings and design options (convenience/privacy).

Figure 4 Willingness to Share Immunity Status with Service Providers (Theatre/Cinema/Gallery) by across settings and design options (convenience/privacy).

For peer review only

For peer review only

Scenario 1 (Visiting the GP for a non-urgent health matter)
You want to book a face-to-face appointment with your GP for a non-urgent matter. A non-urgent matter is one that does not warrant using 111 or 999 NHS services. In order to ensure patient and medical personnel safety, your GP practice has health and safety measures in place requiring patients to prove their COVID-19 (SARS-CoV-2) immunity status when booking a face-to-face appointment. Proof of your immunity status will determine the location and time of the appointment. Now, let's consider two different ways in which this service could be implemented:

> **Option 1A, Convenience:** Your GP practice has the authority to check your immunity status upon booking the appointment by checking the information in your NHS patient electronic health record (you'll have to share your NHS number). There are no additional steps on your behalf.

> **Option 1B, Privacy:** Your GP practice is not authorised to check your immunity status by accessing your NHS electronic health record. Instead, you would need either extract a QR code from your smartphone's NHS app and share it with your GP practice before confirming your booking or presenting a formal paper certificate proving your status.

Scenario 2 (Dining in a restaurant)
You want to go out for a meal at a restaurant, which only has indoor tables. In order to ensure the safety of the staff and the other diners, the restaurant has health and safety measures in place requiring customers to prove their COVID-19 (SARS-CoV-2) immunity status when making a reservation. Proof of your immunity status will enable you to dine at this restaurant. If you choose not to share your status, you would not be able to dine at this restaurant or book a table. Now, let's consider two different ways in which this service could be implemented:

> **Option 2A, Convenience:** The restaurant has the authority to check your status with the NHS upon booking a table or at least 48 hours prior. You would share your NHS number with the restaurant to enable them to check your immunity status. You would then be able to dine at the restaurant at your booked time without any extra steps on your behalf.

> **Option 2B, Privacy:** The restaurant has no authority to check your immunity status with the NHS on your behalf. Instead, you would need either to extract a QR code from your smartphone's NHS app and share it with the restaurant or present a formal paper certificate upon confirming your reservation.

Scenario 3 (Attending a performance in the theatre)
You want to attend a play at your local theatre, this is an indoor event. In order to ensure the safety of the audience, actors and the other staff, the theatre has health and safety measures in place requiring customers to prove their COVID-19 (SARS-CoV-2) immunity status when booking a ticket for a play. Proof of your immunity status will enable you to attend this play. If you choose not to share your status, you would not be able to attend this indoor event on the selected day. Now, let's consider two different ways in which this service could be implemented:

> **Option 3A, Convenience:** The theatre company has the authority to check your status with the NHS upon booking a ticket to a play or at least 48 hours prior. You would share your NHS number with the theatre company to enable them to check your immunity status. You would then be able to attend the play without any extra steps on your behalf.

> **Option 3B, Privacy:** The theatre company has no authority to check your immunity status with the NHS on your behalf. Instead, you would need either to extract a QR code from your smartphone's NHS app and share it with the theatre company or present a formal paper certificate upon confirming your booking or on the day of the play.

For peer review only

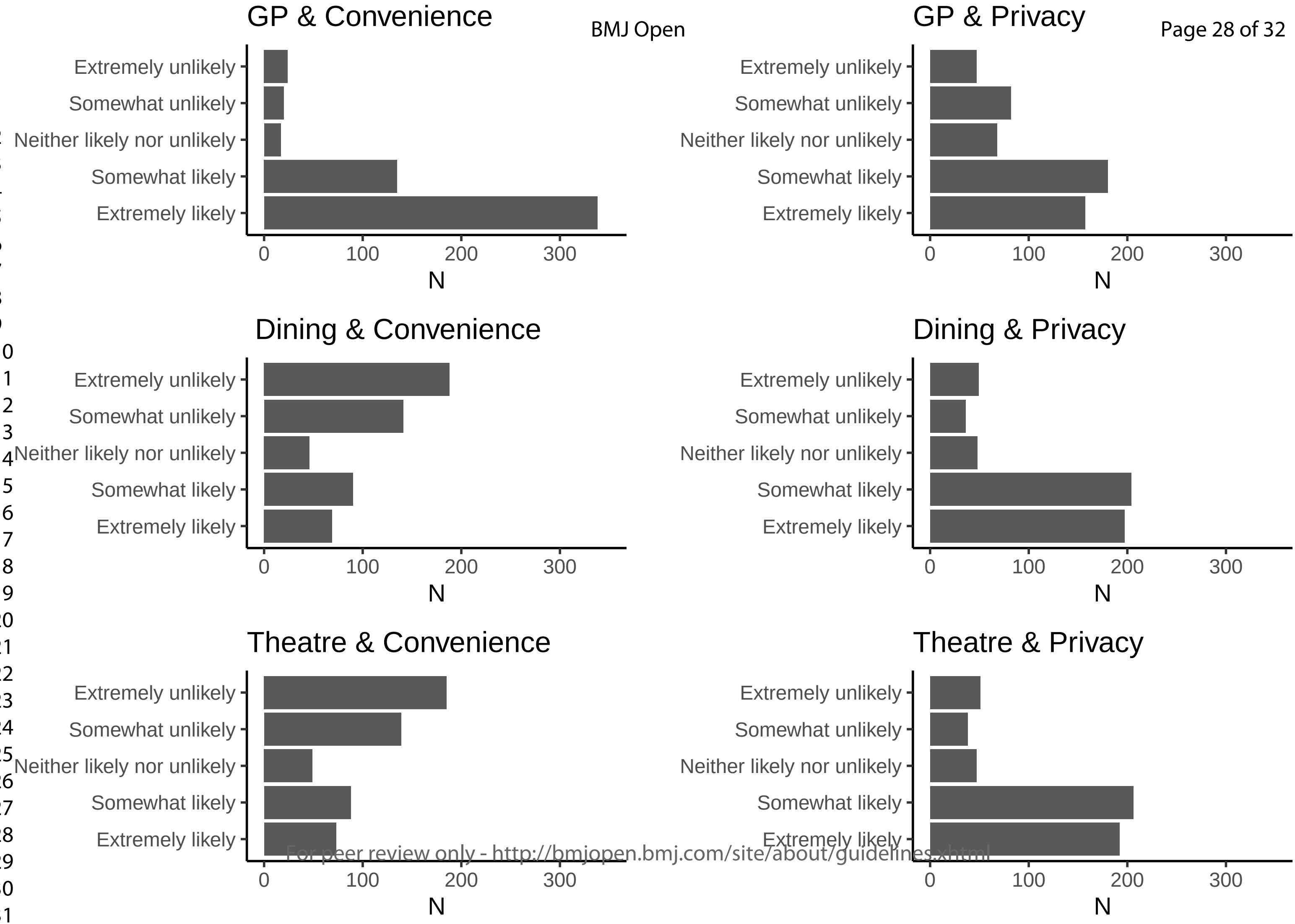

For peer review only - http://bmjopen.bmj.com/site/about/guidelines.xhtml

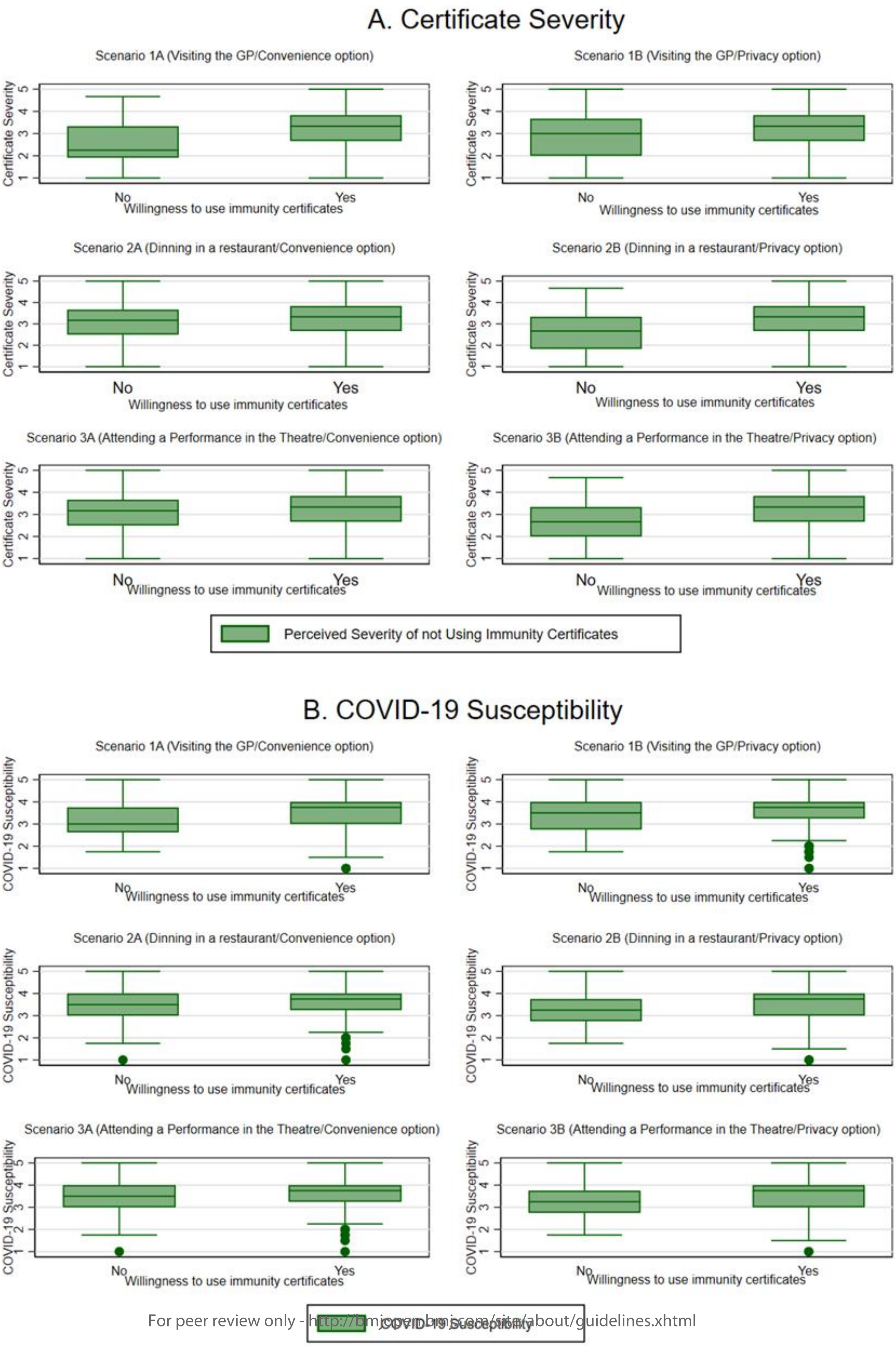
A. Certificate Severity
Scenario 1A (Visiting the GP/Convenience option)
Scenario 1B (Visiting the GP/Privacy option)
Scenario 2A (Dinning in a restaurant/Convenience option)
Scenario 2B (Dinning in a restaurant/Privacy option)
Scenario 3A (Attending a Performance in the Theatre/Convenience option)
Scenario 3B (Attending a Performance in the Theatre/Privacy option)
Certificate Severity
Willingness to use immunity certificates
No
Yes
Perceived Severity of not Using Immunity Certificates
B. COVID-19 Susceptibility
Scenario 1A (Visiting the GP/Convenience option)
Scenario 1B (Visiting the GP/Privacy option)
Scenario 2A (Dinning in a restaurant/Convenience option)
Scenario 2B (Dinning in a restaurant/Privacy option)
Scenario 3A (Attending a Performance in the Theatre/Convenience option)
Scenario 3B (Attending a Performance in the Theatre/Privacy option)
COVID-19 Susceptibility
Willingness to use immunity certificates
No
Yes
COVID-19 Susceptibility

For peer review only

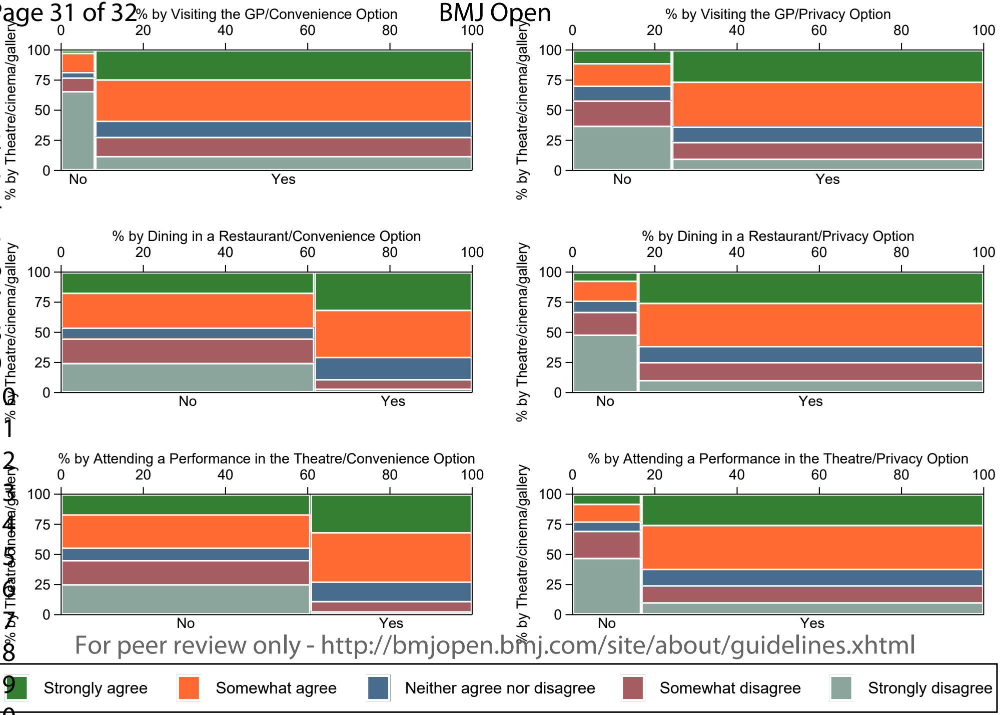

For peer review only - http://bmjopen.bmj.com/site/about/guidelines.xhtml

Supplementary Materials

Supplementary Table 1 Demographic characteristics of sample

		Freq.	Percent	Cum.
**Gender**	Female	277	51.87%	51.87%
	Male	254	47.57%	99.44%
	Self-define	1	0.19%	100%
	Prefer not to say	2	0.37%	99.81%
**Age**	18 – 23	77	14.42%	14.42%
	24 – 29	51	9.55%	23.97%
	30 – 39	95	17.79%	41.76%
	40 – 49	87	16.29%	58.05%
	50 – 59	95	17.79%	75.84%
	60 – 69	109	20.41%	96.25%
	70 or older	20	3.75%	100%
**Ethnicity**	Asian	34	6.37%	6.37%
	Black	20	3.75%	10.11%
	Hispanic/Latino	3	0.56%	10.67%
	Mixed	15	2.81%	13.48%
	Other	8	1.50%	14.98%
	South Asian	12	2.25%	17.23%
	White	442	82.77%	100%
**Region**	East Midlands	42	7.87%	7.87%
	East of England	35	6.55%	14.42%
	London	81	15.17%	29.59%
	Northeast	32	5.99%	35.58%
	Northern Ireland	11	2.06%	37.64%
	Northwest England	58	10.86%	48.50%
	Scotland	37	6.93%	55.43%
	South-East England	87	16.29%	71.72%
	Southwest of England	43	8.05%	79.78%
	Wales	19	3.56%	83.33%
	West Midlands	45	8.43%	91.76%
	Yorkshire and the Humber	44	8.24%	100%
**Area**	Rural	166	31.09%	31.09%
	Urban	368	68.91%	100%
**Accommodation**	Living alone	87	16.29%	16.29%
	Living in shared accommodation	54	10.11%	26.40%
	Living with other family members	382	71.54%	97.94%
	Other	11	2.06%	100%
**Employment**	Employed/Self-employed	340	63.67%	63.67%
	Retired	97	18.16%	81.84%
	Unemployed	97	18.16%	100%
**Education**	A level (or equivalent)	130	24.34%	24.34%
	GCSE (or equivalent)	80	14.98%	39.33%
	Postgraduate degree	95	17.79%	57.12%
	Undergraduate degree	175	32.77%	89.89%
	Vocational	54	10.11%	100%
**Disability**	No	467	87.45%	87.45%
	Prefer not to say	6	1.12%	88.58%
	Yes	61	11.42%	100%
All		*534*	100%	

For peer review only

For peer review only
